# Hot-Carrier Transfer across a Nanoparticle–Molecule Junction: The Importance of Orbital Hybridization and Level Alignment

**DOI:** 10.1021/acs.nanolett.2c02327

**Published:** 2022-10-06

**Authors:** Jakub Fojt, Tuomas P. Rossi, Mikael Kuisma, Paul Erhart

**Affiliations:** † Department of Physics, Chalmers University of Technology, SE-412 96 Gothenburg, Sweden; ‡ Department of Applied Physics, Aalto University, FI-00076 Aalto, Finland; § Department of Physics, Technical University of Denmark, DK-2800 Kongens Lyngby, Denmark

**Keywords:** Hot-carrier, TDDFT, Plasmonic catalysis, Nanoparticles, Adsorption

## Abstract

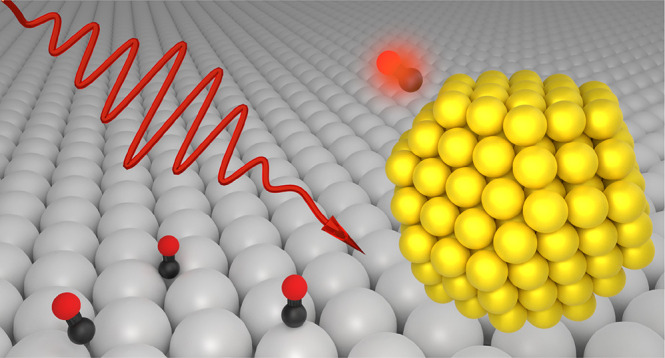

While direct hot-carrier transfer can increase photocatalytic activity, it is difficult to discern experimentally and competes with several other mechanisms. To shed light on these aspects, here, we model from first-principles hot-carrier generation across the interface between plasmonic nanoparticles and a CO molecule. The hot-electron transfer probability depends nonmonotonically on the nanoparticle–molecule distance and can be effective at long distances, even before a strong chemical bond can form; hot-hole transfer on the other hand is limited to shorter distances. These observations can be explained by the energetic alignment between molecular and nanoparticle states as well as the excitation frequency. The hybridization of the molecular orbitals is the key predictor for hot-carrier transfer in these systems, emphasizing the necessity of ground state hybridization for accurate predictions. Finally, we show a nontrivial dependence of the hot-carrier distribution on the excitation energy, which could be exploited when optimizing photocatalytic systems.

Plasmonic metal nanoparticles (NPs) are fundamental components in several emerging technologies, including sensing,^1,2^ light-harvesting,^3^ solar-to-chemical energy conversion,^4−6^ and catalysis.^7−10^ The properties that set these materials apart for these applications are their high surface-to-volume ratios and high optical absorption cross sections at visible frequencies,^11,12^ the latter being due to the presence of a localized surface plasmon (LSP) resonance.^13^ In particular plasmonically driven catalysis is an active research field, addressing important chemical reactions such as ethylene epoxidation, CO oxidation, or NH_3_ oxidation that are catalyzed by illuminating NPs, e.g., of the noble metals Ag,^10,14^ Au,^5,6,15^ or Cu.^8,9^

The LSP, which is a collective electronic excitation, is excited by absorption of light and decays within tens of femtoseconds^4,16−19,19−22^ into a highly nonthermal (usually referred to as “hot”) distribution of electrons and holes.^23−29^ Chemical reactions can then be catalyzed by hot carriers (HCs) transiently populating orbitals of nearby molecules,^4,30^ which can lower reaction barriers.^18^ Two variants of this process can be distinguished. In the *direct* HC transfer process^31^ (also known as chemical interface damping) the LSP decays into an electron–hole pair, where one of the carriers is localized on the reactant molecule and the other on the NP. In the *indirect* HC transfer process both carriers are generated in the NP and at a later time scattered into molecular orbitals. The efficiency and importance of these processes as well as their competition with thermal effects are still a matter of intense debate.^18,32−35^ The direct HC transfer process is promising in terms of efficiency and selectivity^14,30^ and has been studied experimentally,^36^ in theory,^31^ and by computational *ab initio* models.^20,21,37^ Typically the focus lies in understanding HC generation at surfaces,^36,38^ but there has not yet been a detailed account of the dependence of HC transfer on molecular position and orientation and whether there are handles for tuning HC devices to particular molecules in all probable states of thermal motion. Yet these aspects are crucial for direct HC transfer processes, which exhibit an intricate dependence on the hybridization of molecular and surface states as also shown in this work.

In this work, we study plasmon decay and carrier generation across a NP–molecule junction, which is the initial step in the direct HC transfer process. We consider plasmonic Ag, Au, and Cu NPs in combination with a CO molecule, the excitations of which are energetically much higher than the plasmon resonance of any of the NPs considered here. In a real-time time-dependent density functional theory (RT-TDDFT)^39^ framework, we drive the system with an ultrafast laser pulse to induce a plasmon. We simulate the electron dynamics in the system until the plasmon has decayed and then analyze the distribution of carriers over the ground state Kohn–Sham (KS) states. To this end, we employ and extend our analysis methods,^19−21^ mapping out the HC transfer efficiency as a function of the NP–molecule geometry (adsorption site, distance, molecular bond length), excitation energy, and material (Ag, Au, Cu).

We consider the CO molecule in the energetically relevant (111) on-top, (111) face-centered cubic (fcc), (100) hollow and corner sites at a range of distances from the Ag_201_ NP, which has an effective diameter of 15 Å (Supplementary Note S1^40−43^). The plasmon (3.8 eV, Figure 1a) and first optical excitation of CO (14.5 eV, Figure S1) are not resonant, and the optical response of the combined NP+CO system is not strongly dependent on geometry (Figure S2).

**Figure 1:**
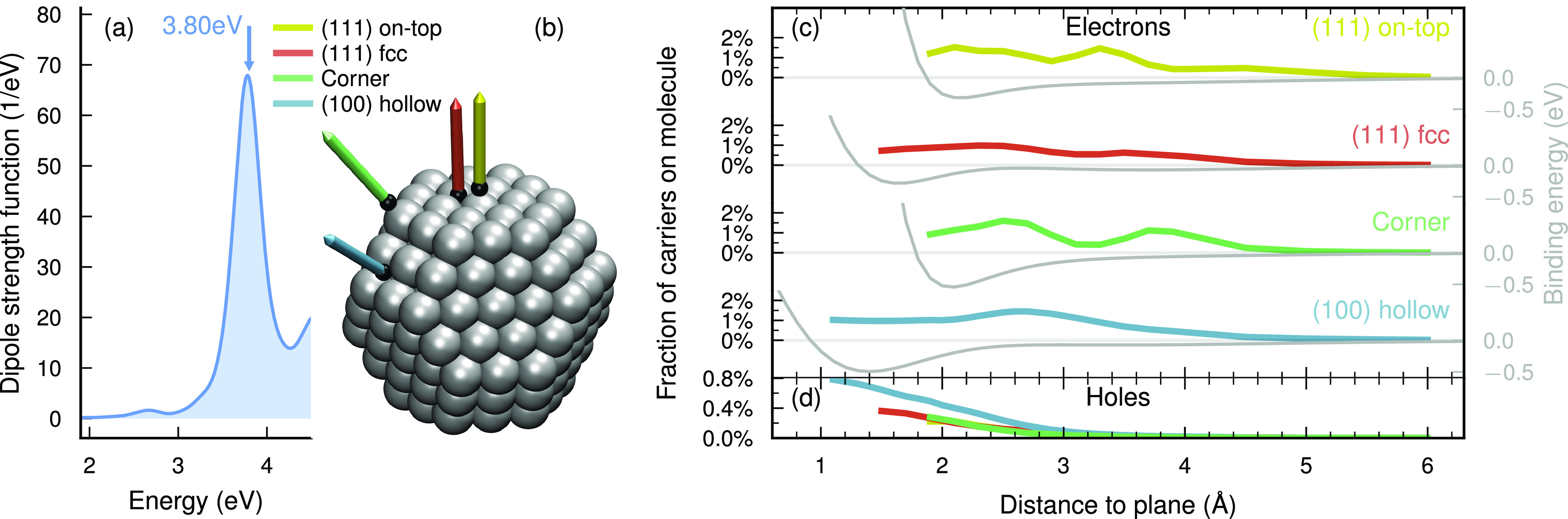
Geometry dependence of HC generation in Ag_201_ NP + CO. (a) Optical spectrum of the bare NP. The frequency 3.8 eV of the driving laser is marked by an arrow above the spectrum. (b) Model of the NP with the axes along which the NP–molecule distance is varied. (c, d) Fractions of generated electrons (c) and holes (d) on the molecule (eq S20) averaged in the span 25–30 fs and binding energies (eq S1) as a function of distance and site.

We drive the Ag NP + CO system with a Gaussian laser pulse with frequency ℏω = 3.8 eV. Within the first tens of femtoseconds a plasmon forms in the NP and decays into resonant excitations, for which the electron–hole energy difference equals ℏω. The plasmon formation and decay process in similar systems have previously been studied in detail by our group^17,19^ and are not covered here.

Varying the NP–CO distance (Figure 1b), we measure the fraction of generated electrons in the molecule after plasmon decay (Figure 1c). Interestingly while the total number of carriers is stabilized already after about 20 fs, for some configurations the fraction of generated electrons in the molecule can exhibit an oscillatory component (Figure S3). This time dependence is deserving of more detailed future studies. Here, we show the average value between 25 and 30 fs which is motivated by the fact that the plasmon has already decayed as the total number of hot carriers has reached a steady value (Figure S3).

While one could expect the fraction of hot electrons on the molecule to decrease monotonically with decreasing wave function overlap at increasing distances, we find this quantity to vary nonmonotonically between 0.5 and 2% over a wide range of distances with several site-specific features. A smooth decay to zero only occurs beyond 4–5 Å. Below this threshold several of the sites feature one or two peaks, including near 2.1 Å and 3.3 Å for the (111) on-top site, 2.7 Å for the (100) hollow site, and 2.7 Å and 3.9 Å for the corner site. Only the (111) fcc site appears relatively featureless.

By contrast, the binding energies depend smoothly on distance and approach zero already at 3–4 Å. The landscape of electron generation on the molecule thus extends further than the features in the potential energy surface and is more sensitive to the underlying shifts in eigenenergies and wave function overlaps. Our findings imply that across-interface HC generation can be effective even at quite long distances (up to 5 Å) from the NP and does not require molecular adsorption.

The fraction of holes generated on the molecule (eq S19), on the other hand, decays smoothly with distance (Figure 1d) reaching a maximum of 0.2–0.8%.

The molecular projected density of states (PDOS) (Figure 2a,d) is a key factor in explaining the rich distance dependence of the across-interface electron generation (Figure 2c,f). The energetic distribution of electrons generated on the molecule (eq S22, Figure 2b,e) clearly mirrors the shape of the molecular PDOS: a single lowest unoccupied molecular orbital (LUMO) level at 2.8 eV at long distances, shifting to lower energies with decreasing distance, eventually splitting into several branches. The mirrored shape is a necessity, as electrons are generated in the unoccupied molecular levels (i.e., where the molecular PDOS is finite); however, two additional factors determine the intensity of the branches. As the transitions (ε_i_ → ε_a_) induced by the plasmon decay are resonant with the pulse frequency (ε_a_ – ε_i_ = ℏω_pulse_ = 3.8 eV), the NP PDOS must align with the molecular PDOS by a constant shift of ℏω_pulse_ (Figure 2, inset). Finally, the strength of the coupling of each electron and hole (via the plasmon decay) is specific to the pair of states (Figure S4). Summarizing the recipe for high across-interface electron generation of energy ε, (1) the molecular PDOS must be large at ε, (2) the NP PDOS must be large at ε – ℏω, and (3) the transition dipole moment between the corresponding NP and molecular states must be sizable.

**Figure 2:**
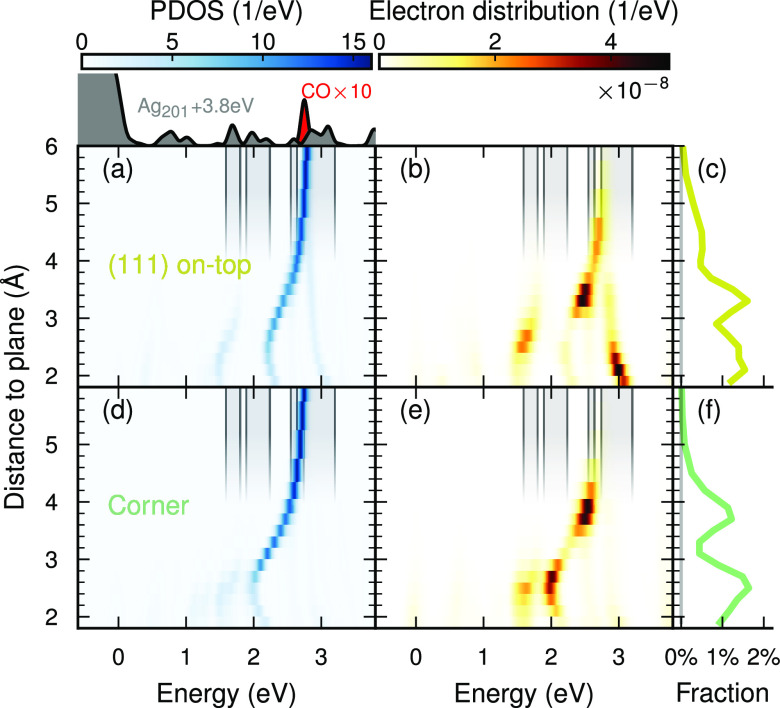
Level alignment between molecular and NP PDOS for (111) on-top (a–c) and corner (d–f) sites. (a, d) Molecular PDOS as a function of distance. The NP PDOS is distance-independent due to the large size of the NP. As the molecule approaches the NP, the LUMO shifts to lower energies, eventually splitting into several branches. The PDOS for the NP and molecule at far separation are indicated above the plot, where the NP PDOS has been shifted by the pulse frequency. Shaded regions correspond to (a selection of) large values in the shifted NP PDOS. (b, e) Electron distribution as a function of distance. (c, f) The fraction of electrons generated in the molecule.

The energetic level alignment is a good descriptor for predicting across-interface HC generation, while surface HC distributions in bare NPs are insufficient for the direct transfer pathway (Supplementary Note S2). PDOSs are usually much simpler to obtain than the transition matrix elements, and we may assess the basic possibility for HC transfer already using the former. The latter (being affected by factors such as wave function overlap and the orbital momentum character of states) is unnecessary for a qualitative description.

Due to the finite line width of the excitation pulse (here the half-width at half-maximum is 0.37 eV), alignment does not have to be exact. In fact, scrutinizing the decomposition of the electrons distribution in terms of the underlying single-particle excitations (Figure S4) shows that the states with the strongest coupling are slightly misaligned with respect to ℏω_pulse_. In the following, we will find that a slight reduction in the excitation frequency leads to a notable increase in the HC transfer probability, as this improves the alignment.

In a similar manner to that of electrons, we can understand the across-interface generation of holes, with the rule that an occupied state ε in the molecule must align with a peak in the NP PDOS at ε + ℏω. As the CO highest occupied molecular orbital (HOMO) level is at −4.8 eV in the free molecule (long distance limit), hole generation is not possible with the pulse frequency 3.8 eV. Transfer is only possible at close distances where hybridized branches of the HOMO and LUMO appear in the region −3.8 eV < ε < 0 eV, beginning at distances around 2.5 Å (Figure S5).

We now extend our study to also include Au and Cu. The s-electrons have nearly identical densities of state (DOSs) in the Ag_201_, Au_201_, and Cu_201_ NPs but the d-band onsets differ (Ag, 3.7 eV; Au, 2.1 eV; Cu, 2.3 eV below the Fermi level; Figure S6). As a consequence of the earlier d-band onset, Au_201_ and Cu_201_ lack the well-defined LSP peak of Ag_201_^44^ (Figure 3a). The binding energy curves are also similar to Ag, with the main difference that the molecule binds more strongly and closer to Cu (Figure S7).

**Figure 3:**
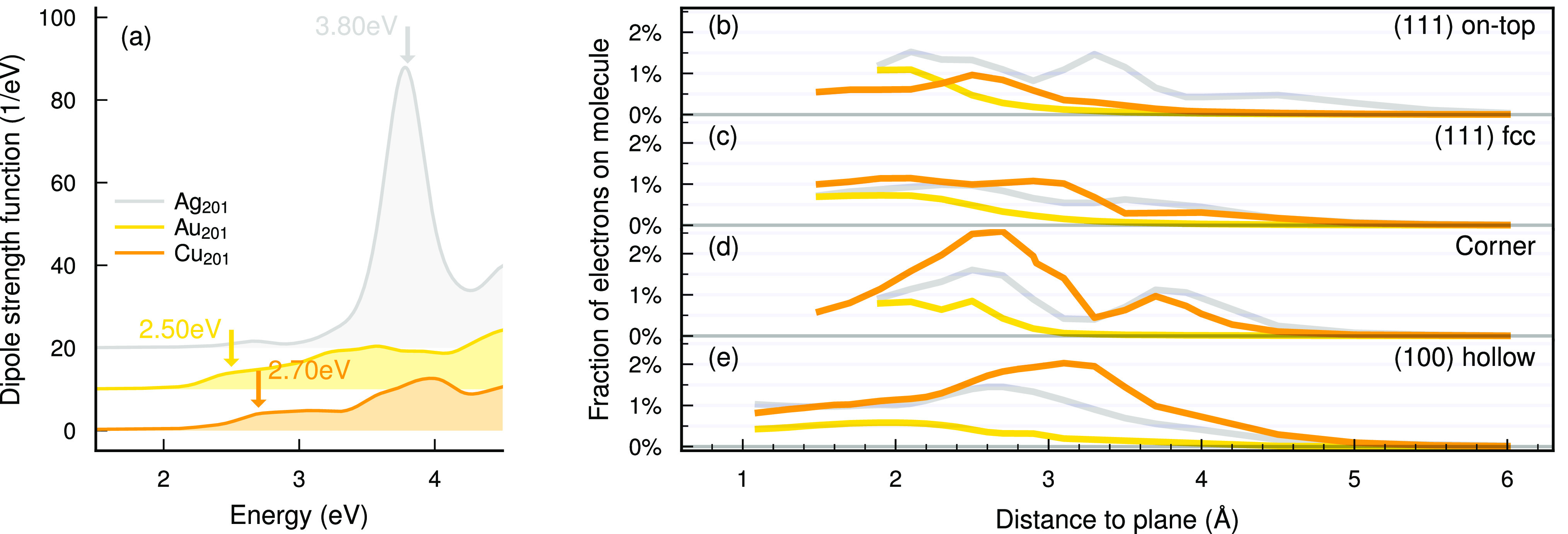
Geometry dependence of electron generation in Ag_201_, Au_201_, and Cu_201_ NPs + CO. (a) Optical spectra of the bare NPs. (b–e) Fractions of generated electrons on the molecule (eq S20) after plasmon decay as a function of distance and site with pulse frequency 3.8 eV (Ag), 2.5 eV (Au), and 2.7 eV (Cu).

We drive the NP + CO with a Gaussian laser pulse (Ag, frequency 3.8 eV; Au, 2.5 eV; Cu, 2.7 eV) and measure the fraction of electrons generated on the molecule (Figure 3b–e). We observe similar trends in Ag and Cu, both exhibiting peaks near 2.1 Å for the (111) on-top site, 2.7 Å for the (100) hollow site, and 2.7 Å and 3.9 Å for the corner site. Only the 3.3 Å peak in the (111) on-top site of Ag lacks a counterpart in Cu. In contrast to Ag and Cu, the Au NP shows smooth trends, without pronounced peaks, of decreasing electron generation on the molecule with increasing distance.

The similarity in electron generation for Ag and Cu can be explained by a similar distance dependence of the molecular orbital hybridization (Figure S5). While the resonance condition is not the same for Ag and Cu (ℏω = 3.8 and 2.7 eV, respectively), the similar energy-spacing between hybridized molecular levels is enough to yield similar electron generation curves. The 3.3 Å peak is the only clear feature that is missing in Cu, the reason being that the molecular orbital is too far from the Fermi level (2.8 eV, to be compared to ℏω = 2.7 eV). The hybridization behavior in Au differs from the behavior in Ag and Cu. At long distances the CO LUMO is further from the Fermi level in Au than in Ag or Cu (due to Au having a higher work function and us considering different bond length of the molecule for each metal), preventing electron generation. As the distance decreases, the orbital hybridizes more strongly, splitting into more branches. As a consequence, at small distances there are more PDOS branches in which electron generation occurs, leading to a smoother distance dependence.

Based on our observations, we should expect the pulse frequency ℏω to act as a handle for tuning the electron generation through the approximate (barring electron–hole coupling) resonance condition ε_a_ – ε_i_ = ℏω. Indeed, the electron generation depends nonmonotonically on both distance and pulse frequency (Figure 4a–c). For example, by lowering the pulse frequency we can avoid the dip in electron generation at 2.9 Å for the Ag (111) on-top site; using ℏω = 3.1 eV, the feature at the same distance instead becomes a maximum. Choosing the pulse frequency appropriately, the fraction of electrons generated on the molecule can be as high as 8.9% for Ag (distance 2.7 Å, ℏω = 3.1 eV), 1.1% for Au (distance 1.9 Å, ℏω = 2.2 eV), and 2.3% for Cu (distance 2.1 Å, ℏω = 2.2 eV). While the electron generation in the Ag and Cu systems shows a complex dependence on pulse frequency and distance, in the case of Au, it is almost monotonic in both dimensions. However, in both end points of the considered pulse frequencies the behavior for Au becomes more interesting; at low frequencies the distance dependence becomes very sharp, as fewer PDOS branches fall into the relevant energy range, and at high frequencies there is electron generation at long distances, due to the free-molecule LUMO falling into the relevant energy range.

**Figure 4:**
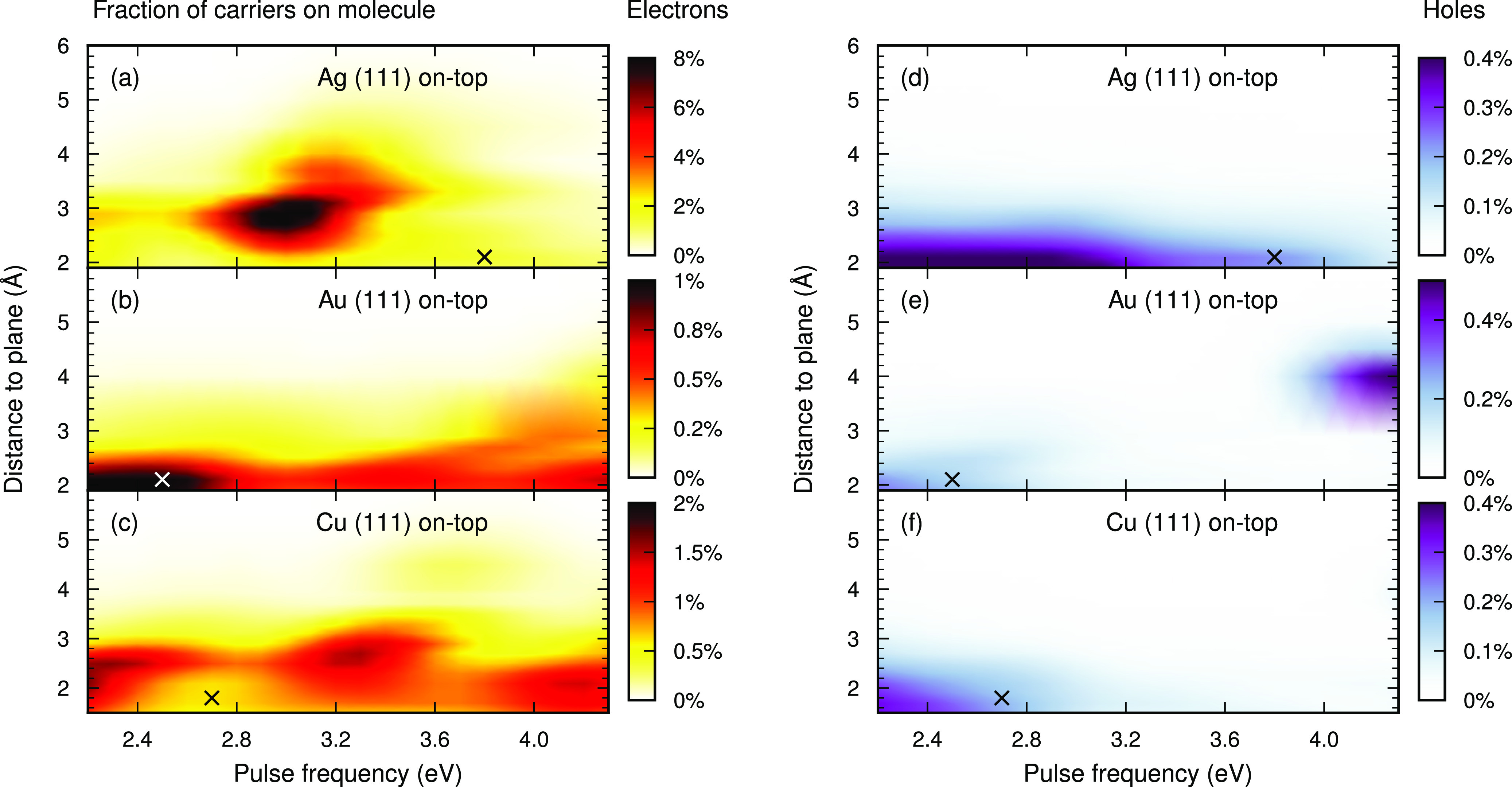
Pulse-frequency dependence of the electron generation in CO. Fraction of generated electrons (a–c) and holes (d–f) on the molecule as a function of distance and pulse frequency for the (111) on-top site in Ag (a, d), Au (b, e), and Cu (c, f). For reference, the crosses in the figure mark the distance corresponding to the adsorption minimum, and the pulse frequency used in Figure 3.

In the case of Ag (Figure 4d) and Cu (Figure 4f), holes are generated at small distances (where hybridized branches of the LUMO orbital are below the Fermi energy; Figure S5) with a weak dependence on pulse frequency. For Au (Figure 4e) hole generation becomes relatively strong at intermediate distances (3–5 Å) and large pulse frequencies. This behavior originates from the HOMO orbital, which in the long-distance limit resides 3.9 eV below the Fermi energy but shifts to lower energies with decreasing distance (i.e., out of the range ε_a_ – ε_i_ = ℏω, ε_a_ > 0, thus limiting hole generation).

The frequency of the exciting light is thus an excellent handle for tuning the fraction of carriers generated on the molecule, which is especially interesting in applications where selectivity is important. In molecules with several orbitals close enough to the Fermi energy to be optically accessible, the generation of electrons in one orbital could be favored over the other. It is, however, important at this stage to remember that changing the pulse frequency also changes the total optical absorption and thus the total number of generated carriers. We therefore also consider the total, pulse-frequency and distance-dependent, amount of electrons generated on the molecule (Figure S8, that is, contrary to before, *without* expressing it as a fraction of electrons generated in the entire NP + CO system). In particular for the Ag NP, which has a very sharp absorption spectrum, the pulse dependence is affected, with a maximum in total electron generation on the molecule occurring using a pulse frequency of 3.6 eV (to be compared to a maximum in the fraction of electrons generated on the molecule at 3.1 eV). As a final note, we point out that it should be possible to simultaneously tune the pulse frequency to the desired resonance condition ε_a_ – ε_i_ = ℏω, and the LSP resonance of the NP (thus the optical absorption) to the pulse frequency, by taking advantage of the fact that the LSP is more sensitive to the size and shape of the NP than, for example, the DOS.

In this study we have investigated the geometry dependence of HC generation across noble metal–molecule interfaces due to plasmon absorption and decay. We have found that typically up to 0.5–3% of all electrons generated in the system end up on the molecule after plasmon decay, even up to distances of 5 Å, which is well before a strong chemical bond can form. By tuning the excitation frequency, we are able to achieve up to 8.9% electrons generated in the molecule, at the expense of a lower absolute amount of electrons generated. These findings suggest that direct HC transfer is a relevant process in plasmon decay and that the process does not require the formation of a strong chemical bond between the molecule and the absorbing medium.

We have also shown that the fraction of generated electrons on the molecule depends on the geometry of the molecule and NP in rather intricate fashion. This geometry dependence can be understood in terms of the energy landscape of hybridized molecular orbitals; as an orbital shifts so it is resonant with certain peaks in the NP PDOS, an increase in the probability for HC transfer can be expected. The distance-dependent behavior of the hybridization differs enough for the various sites so that the carrier generation also differs. For larger NPs, where the DOS between d-band onset and Fermi level is more smeared out, these effects could be less important.

In HC transfer processes the rate of charge carrier generation across the metal–molecule interface is in competition with various loss channels, such as the rates of reemission and scattering and subsequent thermalization of excited carriers with phonons, surfaces, and other carriers.^16,26^ The most important mechanisms in this context are electron–electron and (to a lesser degree) electron–phonon scattering, which are necessary for the HC distribution to thermalize to a Fermi–Dirac distribution. As these loss channels are currently beyond the reach of our calculations, we can view our results as an upper bound on the efficiency of HC transfer, i.e., out of all photon absorption events that occur, up to 0.5–3% (or 8.9% when tuning the excitation frequency) result in electron transfer to the molecule. It is worthwhile to point out that HC generation is a quantized process, where it is very unlikely that there is at one time more than one excited plasmon at a time, under illumination conditions that are realistic for energy-harvesting applications.^26,27^ Each plasmon decays into one electron–hole pair where one carrier can be either in the molecule or in the metal, and we should thus consider our computed fractions as probabilities. We also emphasize that the numbers we report are specific for the NP size and adsorbate coverage considered here.

For the purpose of a quantitative comparison to experimental realizations, it is crucial to consider that while metal states are accurately described with our level of theory,^45^ molecular states are not. We should thus expect transfer maxima to occur for different geometrical configurations, due to slightly shifted (hybridized) molecular orbitals; however, our general conclusions are still valid.

The importance of ground state hybridization for HC transfer implies that theoretical modeling should not be restricted to considering bare metal surfaces, without taking interactions with molecules into account. The distance-dependent hybridization of molecular orbitals should be explicitly taken into account for meaningful predictions. Our results suggest that since already the ground state of the hybridized system is a good descriptor for prediction of HC generation on molecules, rapid screening of candidates of good systems can be performed without conducting expensive real-time simulations.

We close by commenting on the possibilities for tuning HC transfer suggested by the results of this study. HC devices can be designed by tuning the resonance condition to achieve a desired purpose; handles for tuning to a certain molecular orbital are the NP DOS, surface substitutions that affect the hybridization of the orbital, and the frequency of the incoming light. As we have demonstrated, the tuning of the last influences the absorption cross section so that there is a trade-off between high fraction of carriers transferred and high amount of carriers generated in total. It is possible, however, to shift the absorption maximum with a rather small impact on level alignment by modifying the NP shape and size so that there is a maximum in both absorption and transfer. In this way one ought to be able to maximize HC transfer. Furthermore, the sharp LSP resonances of Ag NPs could possibly be utilized in the design of highly selective catalysts that work with broadband (solar) light; if the NP PDOS consists of one particularly strong peak and that peak is resonant with one specific molecular orbital with the frequency of the LSP resonance, then transfer to that specific orbital will be preferred over transfer to other orbitals.

## Software Used

The VASP^46−49^ suite with the projector augmented wave (PAW)^50^ method and the vdW-df-cx^51−54^ exchange correlation (XC) functional was used for the total energy calculations and structure relaxations. The GPAW package^55,56^ with linear combination of atomic orbitals (LCAO) basis sets^57^ and the LCAO-RT-TDDFT implementation^58^ was used for the RT-TDDFT calculations. The Gritsenko–van Leeuwen–van Lenthe–Baerends solid correlation (GLLB-sc)^45,59^ XC-functional, utilizing the Libxc^60^ library, was used in GPAW. The ASE library^61^ was used for constructing and manipulating atomic structures. The NumPy,^62^ SciPy,^63^ and Matplotlib^64^ Python packages and the VMD software^65,66^ were used for processing and plotting data. The Snakemake^67^ package was used for managing the calculation workflow.

## Supporting Information Available

Supporting data for our results in Figures S1–S15 and Supplementary Table S1; details concerning our methodology, the systems under study, and the parameters used in computations provided in Supplementary Notes S1–S4 (PDF)

## Notes

**Data Availability.** The data generated in this study are openly available via Zenodo at https://doi.org/10.5281/zenodo.6524101.

## References

[ref1] Nugroho F. A. A.; Darmadi I.; Cusinato L.; Susarrey-Arce A.; Schreuders H.; Bannenberg L. J.; da Silva Fanta A. B.; Kadkhodazadeh S.; Wagner J. B.; Antosiewicz T. J.; Hellman A.; Zhdanov V. P.; Dam B.; Langhammer C. Metal–Polymer Hybrid Nanomaterials for Plasmonic Ultrafast Hydrogen Detection. Nat. Mater. 2019, 18, 489–495. 10.1038/s41563-019-0325-4. 30936481

[ref2] Darmadi I.; Khairunnisa S. Z.; Tomeček D.; Langhammer C. Optimization of the Composition of PdAuCu Ternary Alloy Nanoparticles for Plasmonic Hydrogen Sensing. ACS Applied Nano Materials 2021, 4, 8716–8722. 10.1021/acsanm.1c01242.

[ref3] Geng X.; Abdellah M.; Bericat Vadell R.; Folkenant M.; Edvinsson T.; Sa J. Direct Plasmonic Solar Cell Efficiency Dependence on Spiro-OMeTAD Li-TFSI Content. Nanomaterials 2021, 11, 3329 10.3390/nano11123329. 34947678 PMC8708565

[ref4] Aslam U.; Rao V. G.; Chavez S.; Linic S. Catalytic Conversion of Solar to Chemical Energy on Plasmonic Metal Nanostructures. Nature Catalysis 2018, 1, 656–665. 10.1038/s41929-018-0138-x.

[ref5] Li R.; Cheng W.-H.; Richter M. H.; DuChene J. S.; Tian W.; Li C.; Atwater H. A. Unassisted Highly Selective Gas-Phase CO2 Reduction with a Plasmonic Au/p-GaN Photocatalyst Using H2O as an Electron Donor. ACS Energy Letters 2021, 6, 1849–1856. 10.1021/acsenergylett.1c00392.

[ref6] DuChene J. S.; Tagliabue G.; Welch A. J.; Cheng W.-H.; Atwater H. A. Hot Hole Collection and Photoelectrochemical CO2 Reduction with Plasmonic Au/p-GaN Photocathodes. Nano Lett. 2018, 18, 2545–2550. 10.1021/acs.nanolett.8b00241. 29522350

[ref7] Zhou L.; Lou M.; Bao J. L.; Zhang C.; Liu J. G.; Martirez J. M. P.; Tian S.; Yuan L.; Swearer D. F.; Robatjazi H.; Carter E. A.; Nordlander P.; Halas N. J. Hot Carrier Multiplication in Plasmonic Photocatalysis. Proc. Natl. Acad. Sci. U. S. A. 2021, 118, e2022109118 10.1073/pnas.2022109118. 33972426 PMC8157927

[ref8] DuChene J. S.; Tagliabue G.; Welch A. J.; Li X.; Cheng W.-H.; Atwater H. A. Optical Excitation of a Nanoparticle Cu/p-NiO Photocathode Improves Reaction Selectivity for CO2 Reduction in Aqueous Electrolytes. Nano Lett. 2020, 20, 2348–2358. 10.1021/acs.nanolett.9b04895. 32134672

[ref9] Hou T.; Chen L.; Xin Y.; Zhu W.; Zhang C.; Zhang W.; Liang S.; Wang L. Porous CuFe for Plasmon-Assisted N2 Photofixation. ACS Energy Letters 2020, 5, 2444–2451. 10.1021/acsenergylett.0c00959.

[ref10] Yamazaki Y.; Kuwahara Y.; Mori K.; Kamegawa T.; Yamashita H. Enhanced Catalysis of Plasmonic Silver Nanoparticles by a Combination of Macro-/Mesoporous Nanostructured Silica Support. J. Phys. Chem. C 2021, 125, 9150–9157. 10.1021/acs.jpcc.1c01669.

[ref11] Bohren C. F. How Can a Particle Absorb More than the Light Incident on It?. Am. J. Phys. 1983, 51, 323–327. 10.1119/1.13262.

[ref12] Langhammer C.; Kasemo B.; Zorić I. Absorption and Scattering of Light by Pt, Pd, Ag, and Au Nanodisks: Absolute Cross Sections and Branching Ratios. J. Chem. Phys. 2007, 126, 194702 10.1063/1.2734550. 17523823

[ref13] Kreibig U.; Vollmer M. Optical Properties of Metal Clusters; Springer Series in Materials Science 25; Springer: Berlin, 1995.

[ref14] Christopher P.; Xin H.; Linic S. Visible-Light-Enhanced Catalytic Oxidation Reactions on Plasmonic Silver Nanostructures. Nat. Chem. 2011, 3, 467–472. 10.1038/nchem.1032. 21602862

[ref15] Saha S.; Yang J.; Masouleh S. S. M.; Botton G. A.; Soleymani L. Hot Hole Direct Photoelectrochemistry of Au NPs: Interband versus Intraband Hot Carriers. Electrochim. Acta 2022, 404, 139746 10.1016/j.electacta.2021.139746.

[ref16] Bernardi M.; Mustafa J.; Neaton J. B.; Louie S. G. Theory and Computation of Hot Carriers Generated by Surface Plasmon Polaritons in Noble Metals. Nat. Commun. 2015, 6, 7044 10.1038/ncomms8044. 26033445 PMC4458868

[ref17] Rossi T. P.; Kuisma M.; Puska M. J.; Nieminen R. M.; Erhart P. Kohn–Sham Decomposition in Real-Time Time-Dependent Density-Functional Theory: An Efficient Tool for Analyzing Plasmonic Excitations. J. Chem. Theory Comput. 2017, 13, 4779–4790. 10.1021/acs.jctc.7b00589. 28862851

[ref18] Zhou L.; Swearer D. F.; Zhang C.; Robatjazi H.; Zhao H.; Henderson L.; Dong L.; Christopher P.; Carter E. A.; Nordlander P.; Halas N. J. Quantifying Hot Carrier and Thermal Contributions in Plasmonic Photocatalysis. Science 2018, 362, 69–72. 10.1126/science.aat6967. 30287657

[ref19] Rossi T. P.; Erhart P.; Kuisma M. Hot-Carrier Generation in Plasmonic Nanoparticles: The Importance of Atomic Structure. ACS Nano 2020, 14, 9963–9971. 10.1021/acsnano.0c03004. 32687311 PMC7458472

[ref20] Kumar P. V.; Rossi T. P.; Kuisma M.; Erhart P.; Norris D. J. Direct Hot-Carrier Transfer in Plasmonic Catalysis. Faraday Discuss. 2019, 214, 189–197. 10.1039/C8FD00154E. 30855061

[ref21] Kumar P. V.; Rossi T. P.; Marti-Dafcik D.; Reichmuth D.; Kuisma M.; Erhart P.; Puska M. J.; Norris D. J. Plasmon-Induced Direct Hot-Carrier Transfer at Metal–Acceptor Interfaces. ACS Nano 2019, 13, 3188–3195. 10.1021/acsnano.8b08703. 30768238

[ref22] Villegas C. E. P.; Leite M. S.; Marini A.; Rocha A. R. Efficient Hot-Carrier Dynamics in near-Infrared Photocatalytic Metals. Phys. Rev. B 2022, 105, 165109 10.1103/PhysRevB.105.165109.

[ref23] Brongersma M. L.; Halas N. J.; Nordlander P. Plasmon-Induced Hot Carrier Science and Technology. Nat. Nanotechnol. 2015, 10, 25–34. 10.1038/nnano.2014.311. 25559968

[ref24] Gong T.; Munday J. N. Materials for Hot Carrier Plasmonics [Invited]. Optical Materials Express 2015, 5, 2501–2512. 10.1364/OME.5.002501.

[ref25] Roman Castellanos L.; Hess O.; Lischner J. Single Plasmon Hot Carrier Generation in Metallic Nanoparticles. Commun. Phys. 2019, 2, 47 10.1038/s42005-019-0148-2.

[ref26] Khurgin J. B. Hot Carriers Generated by Plasmons: Where Are They Generated and Where Do They Go from There?. Faraday Discuss. 2019, 214, 35–58. 10.1039/C8FD00200B. 30806397

[ref27] Khurgin J. B. Fundamental Limits of Hot Carrier Injection from Metal in Nanoplasmonics. Nanophotonics 2020, 9, 453–471. 10.1515/nanoph-2019-0396.

[ref28] Hattori Y.; Meng J.; Zheng K.; Meier de Andrade A.; Kullgren J.; Broqvist P.; Nordlander P.; Sa J. Phonon-Assisted Hot Carrier Generation in Plasmonic Semiconductor Systems. Nano Lett. 2021, 21, 1083–1089. 10.1021/acs.nanolett.0c04419. 33416331 PMC7877730

[ref29] Hawe P.; Silveira V. R. R.; Bericat Vadell R.; Lewin E.; Sa J. Plasmon-Mediated Oxidation Reaction on Au/p-Cu2O: The Origin of Hot Holes. Physchem 2021, 1, 163–175. 10.3390/physchem1020011.

[ref30] Linic S.; Aslam U.; Boerigter C.; Morabito M. Photochemical Transformations on Plasmonic Metal Nanoparticles. Nat. Mater. 2015, 14, 567–576. 10.1038/nmat4281. 25990912

[ref31] Khurgin J. B.; Petrov A.; Eich M.; Uskov A. V. Direct Plasmonic Excitation of the Hybridized Surface States in Metal Nanoparticles. ACS Photonics 2021, 8, 2041–2049. 10.1021/acsphotonics.1c00167.

[ref32] Dubi Y.; Un I. W.; Sivan Y. Thermal Effects – an Alternative Mechanism for Plasmon-Assisted Photocatalysis. Chemical Science 2020, 11, 5017–5027. 10.1039/C9SC06480J. 34122958 PMC8159236

[ref33] Jain P. K. Comment on “Thermal Effects – an Alternative Mechanism for Plasmon-Assisted Photocatalysis” by Y. Dubi, I. W. Un and Y. Sivan, Chem. Sci., 2020, 11, 5017. Chem. Sci. 2020, 11, 9022–9023. 10.1039/D0SC02914A. 34125118 PMC8163434

[ref34] Sivan Y.; Baraban J.; Un I. W.; Dubi Y. Comment on “Quantifying Hot Carrier and Thermal Contributions in Plasmonic Photocatalysis”. Science 2019, 364, eaaw9367 10.1126/science.aaw9367. 31048461

[ref35] Zhou L.; Swearer D. F.; Robatjazi H.; Alabastri A.; Christopher P.; Carter E. A.; Nordlander P.; Halas N. J. Response to Comment on “Quantifying Hot Carrier and Thermal Contributions in Plasmonic Photocatalysis”. Science 2019, 364, eaaw9545 10.1126/science.aaw9545. 31048463

[ref36] Seemala B.; Therrien A. J.; Lou M.; Li K.; Finzel J. P.; Qi J.; Nordlander P.; Christopher P. Plasmon-Mediated Catalytic O2 Dissociation on Ag Nanostructures: Hot Electrons or Near Fields?. ACS Energy Letters 2019, 4, 1803–1809. 10.1021/acsenergylett.9b00990.

[ref37] Ma J.; Gao S. Plasmon-Induced Electron–Hole Separation at the Ag/TiO _2_ (110) Interface. ACS Nano 2019, 13, 13658–13667. 10.1021/acsnano.9b03555. 31393703

[ref38] Sundararaman R.; Narang P.; Jermyn A. S.; Goddard W. A. III; Atwater H. A. Theoretical Predictions for Hot-Carrier Generation from Surface Plasmon Decay. Nat. Commun. 2014, 5, 5788 10.1038/ncomms6788. 25511713 PMC4284641

[ref39] Yabana K.; Bertsch G. F. Time-Dependent Local-Density Approximation in Real Time. Phys. Rev. B 1996, 54, 4484–4487. 10.1103/PhysRevB.54.4484. 9986402

[ref40] Gajdo M.; Eichler A.; Hafner J. CO Adsorption on Close-Packed Transition and Noble Metal Surfaces: Trends from Ab Initio Calculations. J. Phys.: Condens. Matter 2004, 16, 1141–1164. 10.1088/0953-8984/16/8/001.

[ref41] Moler E. J.; Kellar S. A.; Huff W. R. A.; Hussain Z.; Chen Y.; Shirley D. A. Spatial Structure Determination of (√3×√3)R30° and (1.5×1.5)R18° CO orCu(111) Using Angle-Resolved Photoemission Extended Fine Structure. Phys. Rev. B 1996, 54, 10862–10868. 10.1103/PhysRevB.54.10862. 9984884

[ref42] Hirschmugl C. J.; Williams G. P.; Hoffmann F. M.; Chabal Y. J. Adsorbate-Substrate Resonant Interactions Observed for Co on Cu(100) and (111) in the Far-Ir Using Synchrotron Radiation. J. Electron Spectrosc. Relat. Phenom. 1990, 54 (55), 109–114. 10.1016/0368-2048(90)80203-M.

[ref43] Raval R.; Parker S. F.; Pemble M. E.; Hollins P.; Pritchard J.; Chesters M. A. FT-rairs, Eels and Leed Studies of the Adsorption of Carbon Monoxide on Cu(111). Surf. Sci. 1988, 203, 353–377. 10.1016/0039-6028(88)90088-X.

[ref44] Cazalilla M. A.; Dolado J. S.; Rubio A.; Echenique P. M. Plasmonic Excitations in Noble Metals: The Case of Ag. Phys. Rev. B 2000, 61, 8033–8042. 10.1103/PhysRevB.61.8033.

[ref45] Kuisma M.; Ojanen J.; Enkovaara J.; Rantala T. T. Kohn-Sham Potential with Discontinuity for Band Gap Materials. Phys. Rev. B 2010, 82, 115106 10.1103/PhysRevB.82.115106.

[ref46] Kresse G.; Hafner J. Ab Initio Molecular Dynamics for Liquid Metals. Phys. Rev. B 1993, 47, 558–561. 10.1103/PhysRevB.47.558. 10004490

[ref47] Kresse G.; Furthmuller J. Efficient Iterative Schemes for Ab Initio Total-Energy Calculations Using a Plane-Wave Basis Set. Phys. Rev. B 1996, 54, 11169–11186. 10.1103/PhysRevB.54.11169. 9984901

[ref48] Kresse G.; Furthmuller J. Efficiency of Ab-Initio Total Energy Calculations for Metals and Semiconductors Using a Plane-Wave Basis Set. Comput. Mater. Sci. 1996, 6, 15–50. 10.1016/0927-0256(96)00008-0. 9984901

[ref49] Kresse G.; Joubert D. From Ultrasoft Pseudopotentials to the Projector Augmented-Wave Method. Phys. Rev. B 1999, 59, 1758–1775. 10.1103/PhysRevB.59.1758.

[ref50] Blochl P. E. Projector Augmented-Wave Method. Phys. Rev. B 1994, 50, 17953–17979. 10.1103/PhysRevB.50.17953. 9976227

[ref51] Berland K.; Hyldgaard P. Exchange Functional That Tests the Robustness of the Plasmon Description of the van Der Waals Density Functional. Phys. Rev. B 2014, 89, 035412 10.1103/PhysRevB.89.035412.

[ref52] Klimeš J.; Bowler D. R.; Michaelides A. Chemical Accuracy for the van Der Waals Density Functional. J. Phys.: Condens. Matter 2009, 22, 022201 10.1088/0953-8984/22/2/022201. 21386245

[ref53] Klimeš J.; Bowler D. R.; Michaelides A. Van Der Waals Density Functionals Applied to Solids. Phys. Rev. B 2011, 83, 195131 10.1103/PhysRevB.83.195131.

[ref54] Roman-Perez G.; Soler J. M. Efficient Implementation of a van Der Waals Density Functional: Application to Double-Wall Carbon Nanotubes. Phys. Rev. Lett. 2009, 103, 096102 10.1103/PhysRevLett.103.096102. 19792809

[ref55] Mortensen J. J.; Hansen L. B.; Jacobsen K. W. Real-Space Grid Implementation of the Projector Augmented Wave Method. Phys. Rev. B 2005, 71, 035109 10.1103/PhysRevB.71.035109.

[ref56] Enkovaara J.; et al. Electronic Structure Calculations with GPAW: A Real-Space Implementation of the Projector Augmented-Wave Method. J. Phys.: Condens. Matter 2010, 22, 253202 10.1088/0953-8984/22/25/253202. 21393795

[ref57] Larsen A. H.; Vanin M.; Mortensen J. J.; Thygesen K. S.; Jacobsen K. W. Localized Atomic Basis Set in the Projector Augmented Wave Method. Phys. Rev. B 2009, 80, 195112 10.1103/PhysRevB.80.195112.

[ref58] Kuisma M.; Sakko A.; Rossi T. P.; Larsen A. H.; Enkovaara J.; Lehtovaara L.; Rantala T. T. Localized Surface Plasmon Resonance in Silver Nanoparticles: Atomistic First-Principles Time-Dependent Density-Functional Theory Calculations. Phys. Rev. B 2015, 91, 115431 10.1103/PhysRevB.91.115431.

[ref59] Gritsenko O.; van Leeuwen R.; van Lenthe E.; Baerends E. J. Self-Consistent Approximation to the Kohn-Sham Exchange Potential. Phys. Rev. A 1995, 51, 1944 10.1103/PhysRevA.51.1944. 9911804

[ref60] Lehtola S.; Steigemann C.; Oliveira M. J. T.; Marques M. A. L. Recent Developments in Libxc — A Comprehensive Library of Functionals for Density Functional Theory. SoftwareX 2018, 7, 1–5. 10.1016/j.softx.2017.11.002.

[ref61] Larsen A. H.; et al. The Atomic Simulation Environment—a Python Library for Working with Atoms. J. Phys.: Condens. Matter 2017, 29, 273002 10.1088/1361-648X/aa680e. 28323250

[ref62] Harris C. R.; et al. Array Programming with NumPy. Nature 2020, 585, 357–362. 10.1038/s41586-020-2649-2. 32939066 PMC7759461

[ref63] Virtanen P.; et al. SciPy 1.0: Fundamental Algorithms for Scientific Computing in Python. Nat. Methods 2020, 17, 261–272. 10.1038/s41592-019-0686-2. 32015543 PMC7056644

[ref64] Hunter J. D. Matplotlib: A 2D Graphics Environment. Computing in Science Engineering 2007, 9, 90–95. 10.1109/MCSE.2007.55.

[ref65] Humphrey W.; Dalke A.; Schulten K. VMD: Visual Molecular Dynamics. J. Mol. Graphics 1996, 14, 33–38. 10.1016/0263-7855(96)00018-5. 8744570

[ref66] Stone J. An Efficient Library for Parallel Ray Tracing and Animation. M.Sc. Thesis, Computer Science Department, University of Missouri-Rolla, 1998.

[ref67] Molder F.; Jablonski K. P.; Letcher B.; Hall M. B.; Tomkins-Tinch C. H.; Sochat V.; Forster J.; Lee S.; Twardziok S. O.; Kanitz A.; Wilm A.; Holtgrewe M.; Rahmann S.; Nahnsen S.; Koster J. Sustainable Data Analysis with Snakemake. F1000Research 2021, 10, 33 10.12688/f1000research.29032.2. 34035898 PMC8114187

