# Supplementary material for: Hot-Carrier Transfer across a Nanoparticle–Molecule Junction: The Importance of Orbital Hybridization and Level Alignment
Source: Nano Lett. 2022 Oct 6;22(21):8786–92. doi: 10.1021/acs.nanolett.2c02327 (PMC9650767; doi:10.1021/acs.nanolett.2c02327)
Supplement: Supplementary file 1 — nl2c02327_si_001.pdf [file nl2c02327_si_001.pdf]

Supporting Information

Hot-carrier transfer across a nanoparticle-molecule junction: The importance of orbital hybridization and level alignment

Jakub Fojt[1], Tuomas P. Rossi[2], Mikael Kuisma[3], and Paul Erhart[1]

[1] *Department of Physics, Chalmers University of Technology, SE-412 96 Gothenburg, Sweden*
[2] *Department of Applied Physics, Aalto University, FI-00076 Aalto, Finland*
[3] *Department of Physics, Technical University of Denmark, DK-2800 Kongens Lyngby, Denmark*

Contents

Supplementary Figures **2**

S1. Absorption spectrum of free CO molecule along its bond axis . . . 2
S2. Frequency and width of Ag_{201} LSP . . . 2
S3. HC generation in Ag_{201} with the CO in (111) on-top configuration. . . . 3
S4. Decomposition of electrons generated on the molecule in electrons and holes, depending on pulse frequency . . . 4
S5. Projected density of states for the molecule, in the combined system . . . 5
S6. Density of states of Ag_{201}, Au_{201}, and Cu_{201} . . . 6
S7. Binding energies of the combined CO + NP system as a function of site and distance . . 6
S8. Pulse-frequency dependence of the electron generation in CO . . . 7
S9. Level alignment between the projected densities of state of the NP and molecule for Ag_{201} 8
S10. Binding energies of CO + Ag_{201} under constrained relaxation . . . 9
S11. Carrier generation on the molecule after plasmon decay . . . 10
S12. Energy distribution of electrons generated on the molecule and on the NP corner site that the molecule approaches . . . 11
S13. Alternative formulations of electron distribution in molecule after plasmon decay 12
S14. Carrier generation depending on occupation number smearing . . . 13
S15. Level alignment between the projected densities of state of the NP and molecule for Au_{201} and Cu_{201} . . . 14

Supplementary Tables **15**

S1. Summary of binding energies, vibrational frequencies and associated bond distances . . . 15

Supplementary Notes **16**

S1. Geometry of atomic structures . . . 16
S2. Comparison of across-interface electron generation to surface electron distribution 16
S3. Methodology . . . 17
S4. Computational details . . . 19

Supplementary References **20**

Supplementary Figures

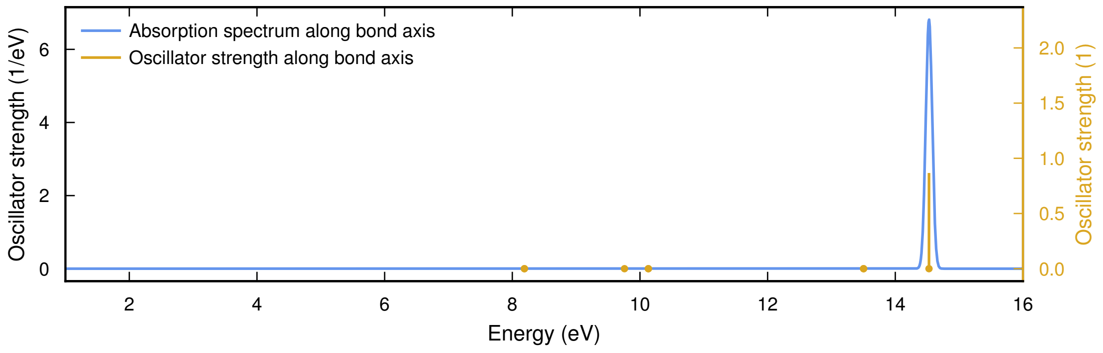

Figure S1: **Absorption spectrum of free CO molecule along its bond axis.** The free CO molecule (bond length 1.144 Å) has its first excited state at 8.19 eV. This is an optically dark state that corresponds to the HOMO-LUMO transition. The first bright state is 14.53 eV. The transitions were obtained by linear response TDDFT calculations in GPAW with the xc-functional PBE.

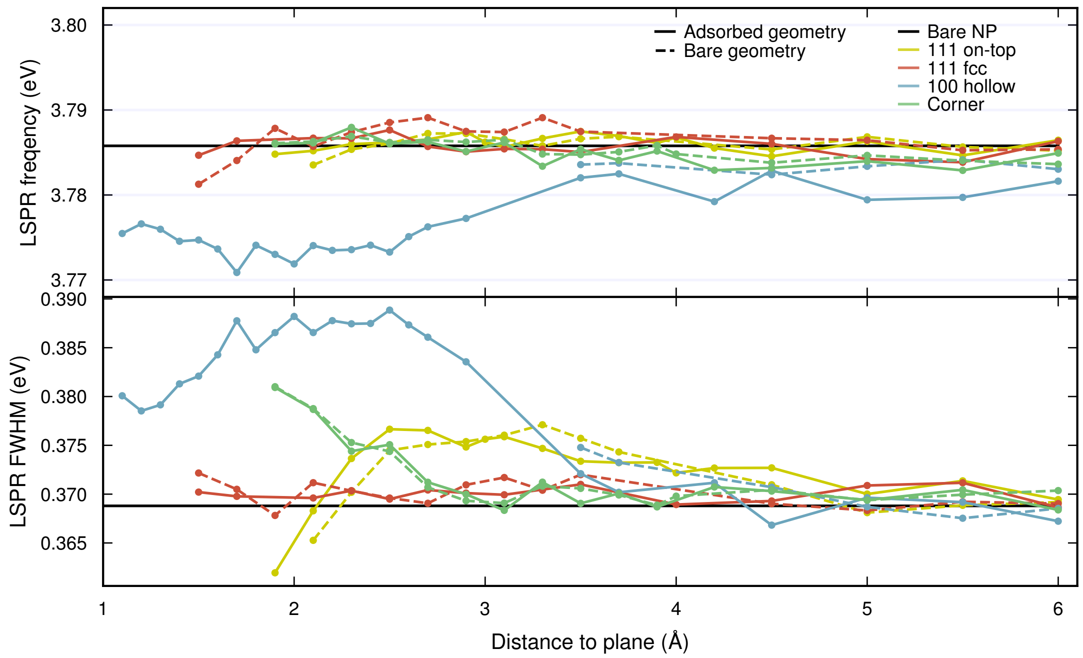

Figure S2: **Frequency and width of Ag_{201} LSP.** The bare NP has a LSP frequency close to 3.8 eV (determined by fitting a Lorentizan function to the spectrum). Adding the CO molecule shifts the LSP frequency by less than 15 meV for the 100 hollow site, and less than 5 meV for the other sites.

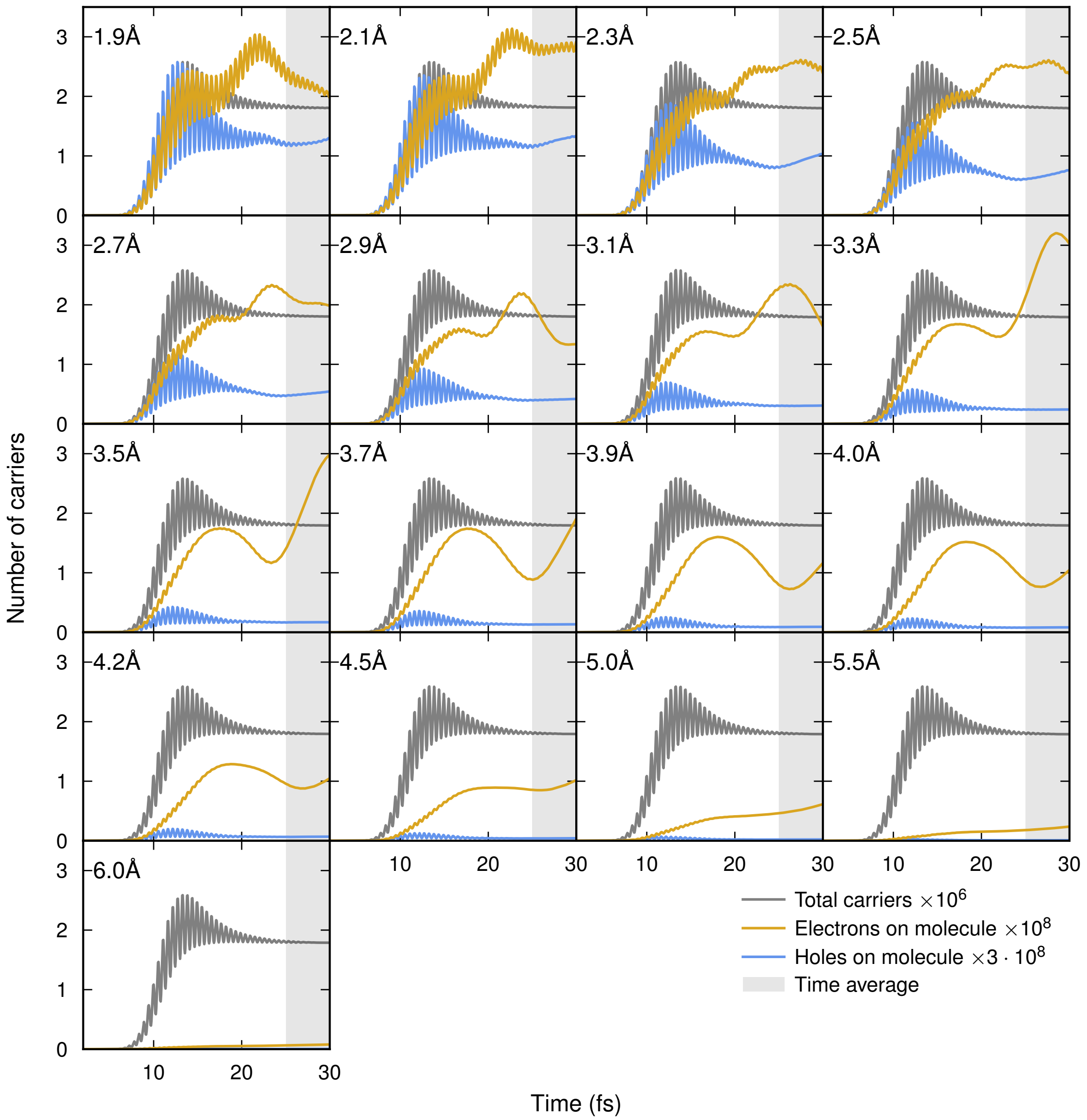

Figure S3: **HC generation in Ag_{201} with the CO in (111) on-top configuration..** The total amount of carriers in the NP + CO, the number of electrons in the CO and the number of holes in the CO are shown as a function of time after excitation with pulse of frequency 3.8 eV. While the total number of carriers in the system stabilizes before the end of the simulation, the number of electrons (in particular) and holes exhibits some oscillations. The oscillations stem from the dynamics between several electron-hole excitations between hybridized NP-molecule states. We note that an apparent maximum occurs in the number of transferred electrons close to the end of the simulation, and that this maximum occurs faster with closer distances (stronger coupling between NP and molecular states). The time span in which time averages are taken elsewhere in the paper (25 to 30 fs) is highlighted in the figure. For all but the intermediate distances, taking the average yields similar results to taking the values at the end of the simulation.

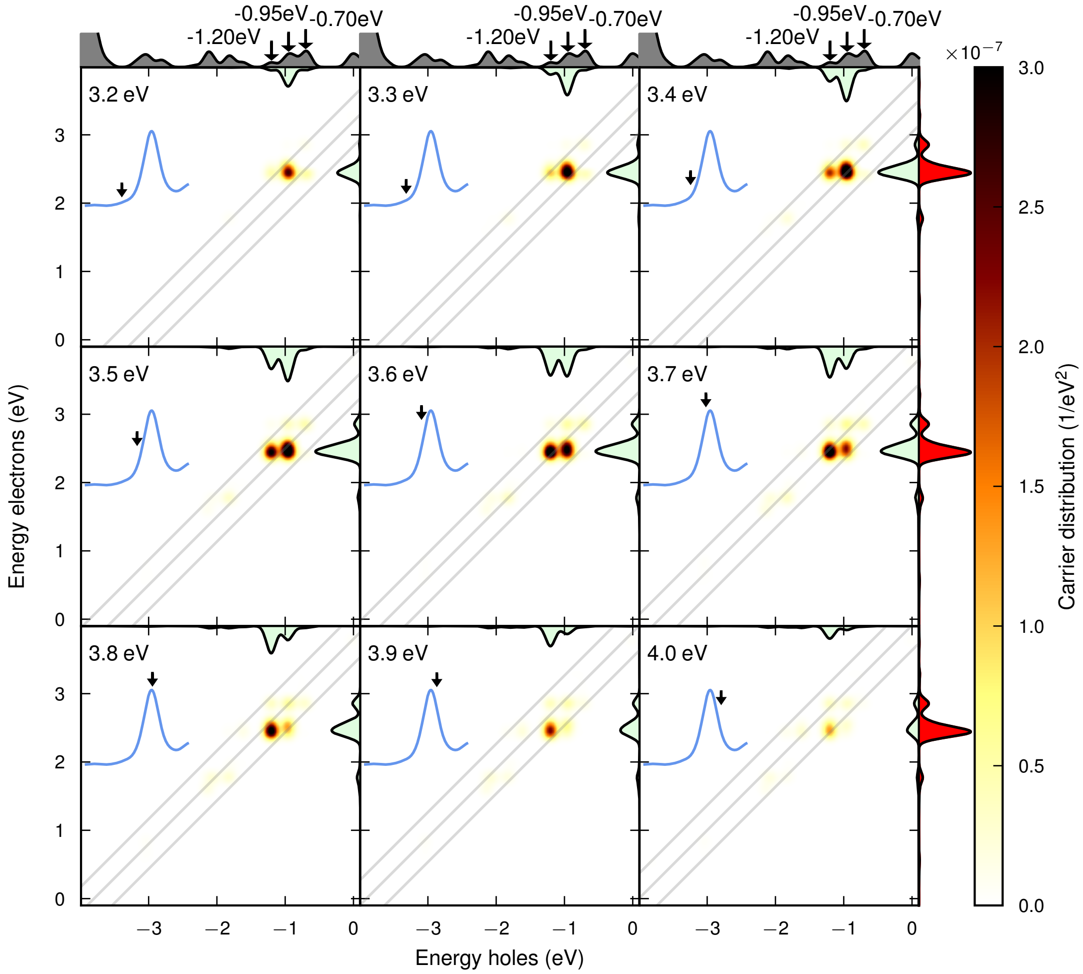

Figure S4: **Decomposition of electrons generated on the molecule in electrons and holes, depending on pulse frequency.** The quantity $\sum_{ia} M_{ia} w^{(\mathrm{mol})}_{aa'} \delta(\varepsilon - \varepsilon_a)\delta(\varepsilon - \varepsilon_i)$ where $M_{ia} = \sum_{a'}^{f_i > f_a, f_i > f_{a'}} (q_{ia}q_{ia'} + p_{ia}p_{ia'})$ shows a map between holes (in the combined system NP+CO) and electrons in the CO molecule, for a particular geometry (Ag_{201} (111) on-top distance 3.3 Å) at 30 fs in the simulation. The map with hole/electron dependence integrated out $(\sum_{ia} M_{ia} w^{(\mathrm{mol})}_{aa'} \delta(\varepsilon - \varepsilon_a) / \sum_{ia} M_{ia} w^{(\mathrm{mol})}_{aa'} \delta(\varepsilon - \varepsilon_i))$ is shown on the inner axes in a fixed scale. DOS of the combined system and PDOS of the molecule are shown on the outer axes. The inset of the optical spectrum shows the pulse frequency used. The electron transfer involves primarily occupied states at three energies (-1.2, -0.95 and -0.7 eV) and unoccupied states in the molecule at three levels. As involved states need to be resonant ($\varepsilon_a - \varepsilon_i = \hbar\omega_{\mathrm{pulse}}$, indicated by the middle diagonal line, while the outer diagonal lines indicate the half-width at half-maximum of the pulse 0.37 eV) the contribution of each state varies with pulse frequency. Maximum electron transfer is achieved at 3.5-3.6 eV, slightly off the LSP resonance, when the states at -1.2 and -0.95 eV are resonant with the main LUMO branch. The former states occupied states are thus interacting more strongly with the transfer to the LUMO than the states at -0.7 eV.

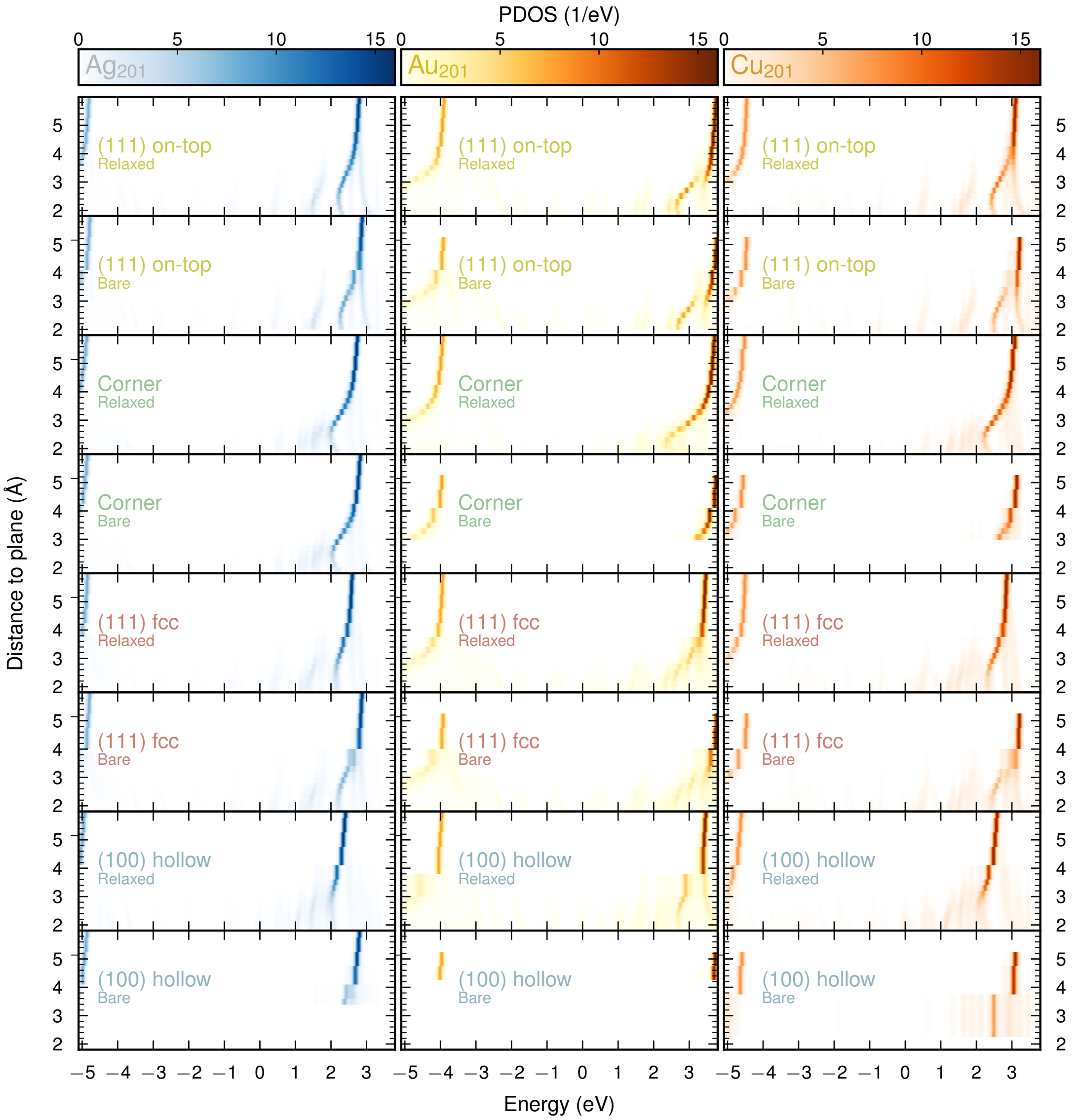

Figure S5: **Projected density of states for the molecule, in the combined system.** As the molecule is brought closer to the NP, the HOMO and LUMO orbitals shift to lower energies (distance 4 to 6 Å) and hybridize, splitting up into several branches (distances smaller than 4 Å).

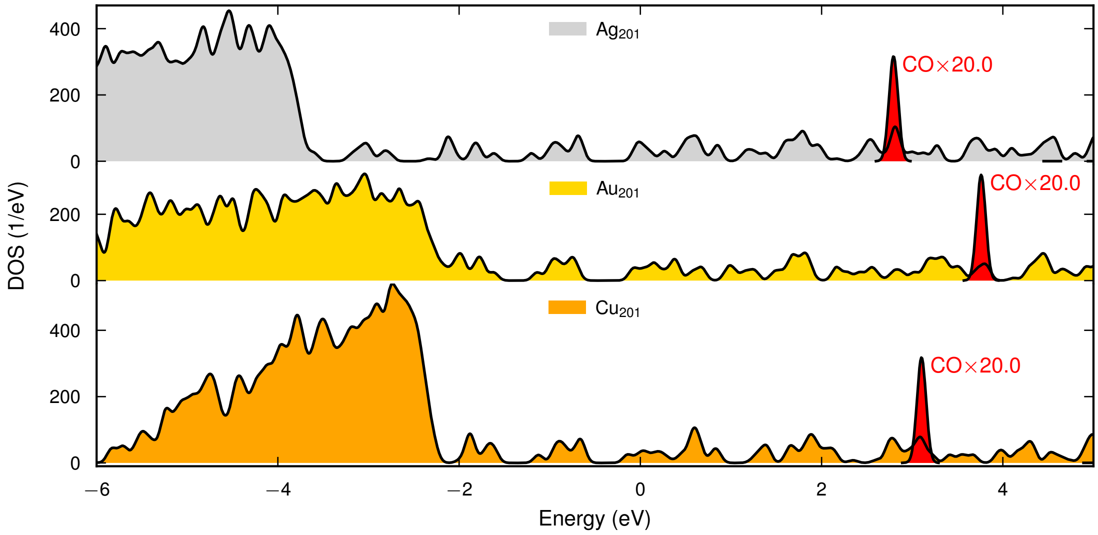

Figure S6: **Density of states of Ag_{201}, Au_{201}, and Cu_{201}.** The DOS is shown relative to Fermi level, and the PDOS of the molecule at far distances is included in the figure, showing its alignment to the different metals.

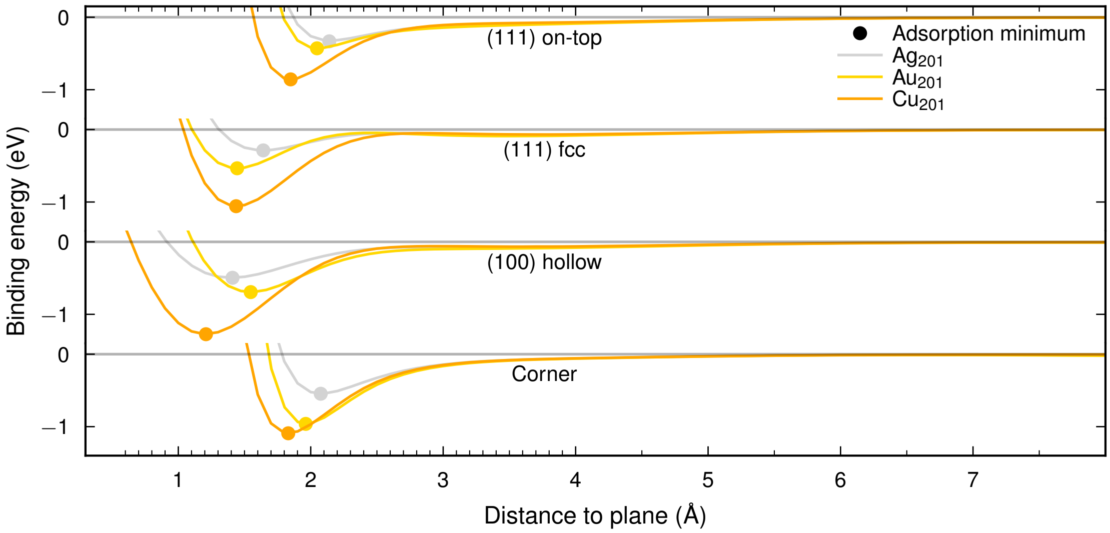

Figure S7: **Binding energies of the combined CO + NP system as a function of site and distance.** Energies are computed in VASP using the vdW-cx-df xc-functional. The molecule is rigidly displaced from its adsorption minimum without allowing neither the bond length nor the NP to relax. The binding energy is defined $E^{(\mathrm{site})}_{\mathrm{bind}}(d) = E^{(\mathrm{site})}(d) - E^{(\mathrm{site})}_{\mathrm{NP}} - E^{(\mathrm{site})}_{\mathrm{mol}}$ where $E^{(\mathrm{site})}_{\mathrm{NP}}$ ($E^{(\mathrm{site})}_{\mathrm{mol}}$) is the energy of the system, in its adsorbed configuration, without the molecule (NP). Ticks in the distance axis mark points distances sampled.

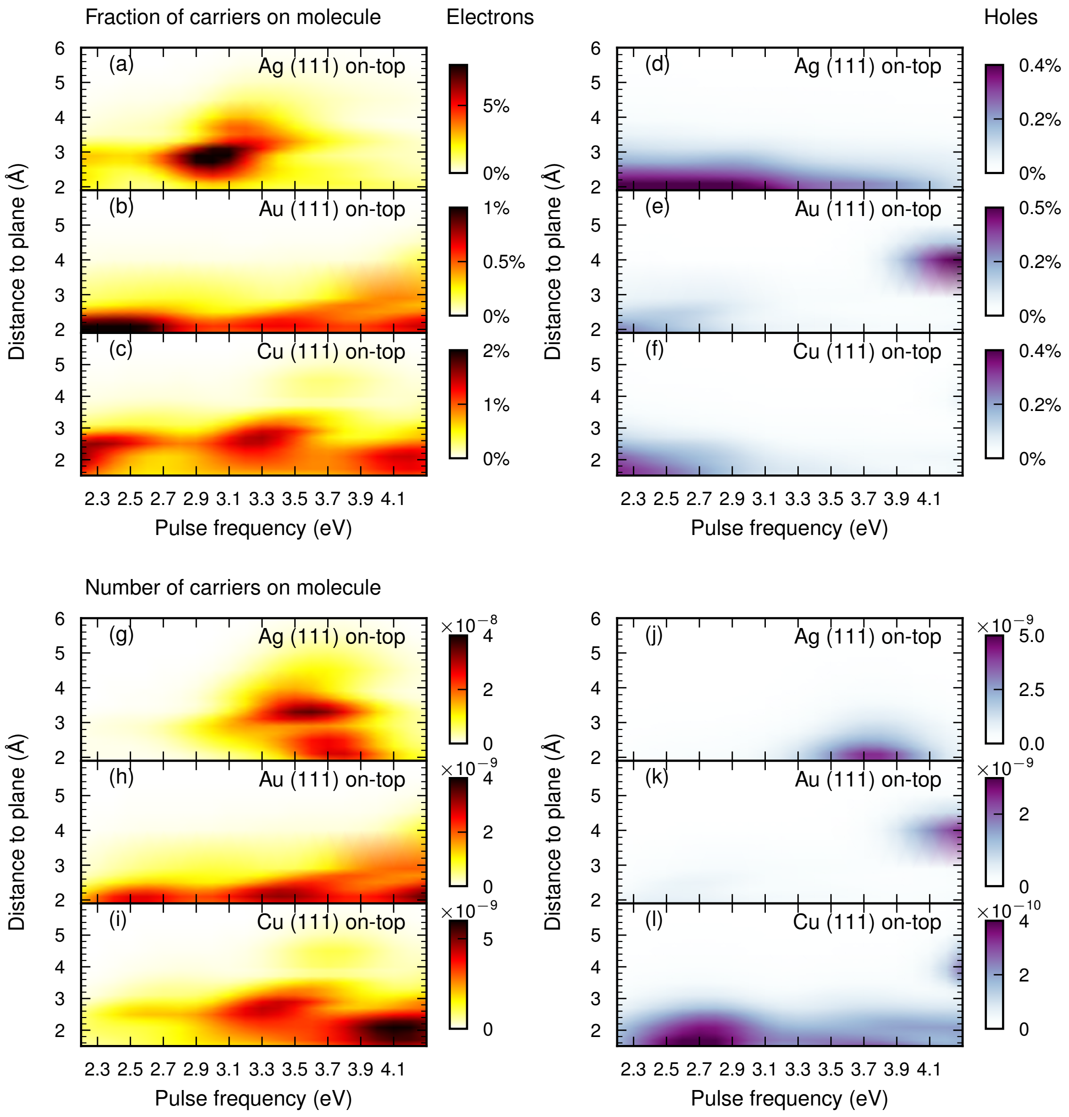

Figure S8: **Pulse-frequency dependence of the electron generation in CO.** The amount of electrons (a-c, g-i) and holes (d-f, j-l) generated on the molecule is expressed as a fraction of carriers (a-f; number of carriers on molecule divided by number of carriers in the combined system) and as a number (g-l). The numbers of generated carriers are computed as the average between 25 and 30 fs in the simulation.

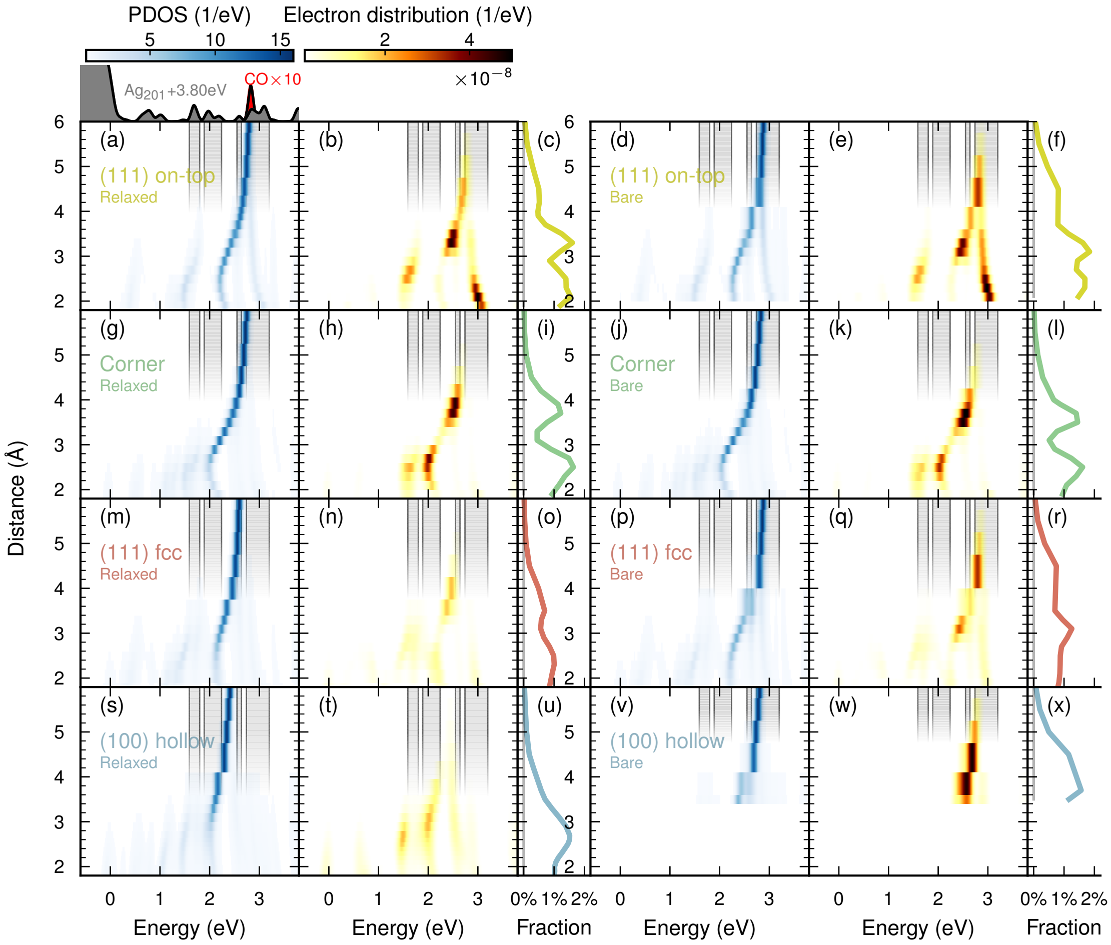

Figure S9: **Level alignment between the projected densities of state of the NP and molecule for Ag_{201}.** "Relaxed" refers to the combined system that has been relaxed in the adsorption minimum, and the molecule then shifted. "Bare" refers to the NP being configured as the free NP and the CO with the bond length of the free CO molecule. For the two types of geometries, the molecular PDOS differs primarily by a constant shift of the LUMO orbital energy (most apparent for the (111) fcc and (100) hollow sites). The shift in PDOS changes the resonance condition, and thus the electron distribution on the molecule and fraction of electrons generated on the molecule. The electron distributions are computed as the average between 25 and 30 fs in the simulation for the Ag_{201} (111) on-top configuration.

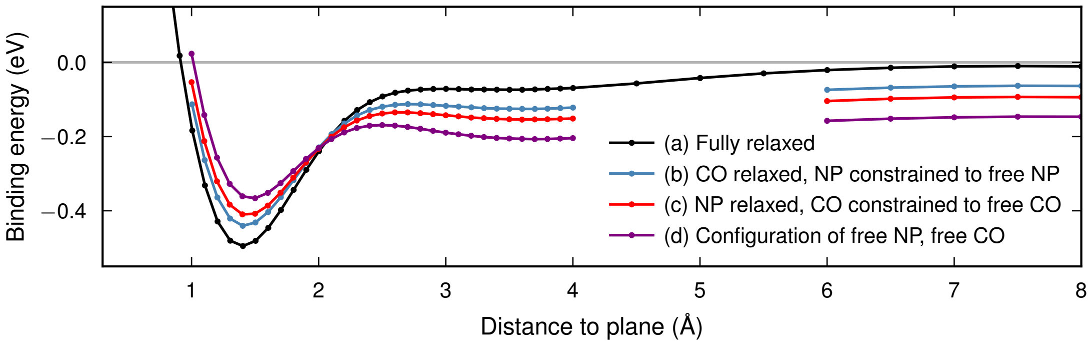

Figure S10: **Binding energies of CO + Ag_{201} under constrained relaxation.** The binding energy is defined $E_{\text{bind}}^{(\text{site})}(d) = E^{(\text{site})}(d) - E_{\text{NP}}^{(\text{site})} - E_{\text{mol}}^{(\text{site})}$ where $E_{\text{NP}}^{(\text{site})}$ ($E_{\text{mol}}^{(\text{site})}$) is the energy of the system, in its adsorbed configuration, without the molecule (NP). Binding energies are computed by rigidly displacing the CO along a line from the (111) on-top site of the NP, from 4 different starting positions. The starting positions are:
(a) The combined system (NP and CO) is fully relaxed in the adsorption minimum (same as in Fig. S7.
(b) The combined system is relaxed with a constraint: all Ag atoms are fixed in the positions of the free NP.
(c) The combined system is relaxed with a constraint: the CO bond length is fixed to the bond length of the free molecule.
(d) The combined system is not relaxed. The configuration of a free NP and free molecule are used.
Of the 4 options fully relaxing the system (a) gives the lowest energies close to the adsorption minimum, but the highest in the long-distance limit, which is expected. Taking the free NP and free molecule configurations (d) gives the highest energies close to the adsorption minimum and the lowest in the long-distance limit. The relaxation of the molecule only (b; the bond length increases compared to the bond length of free CO) and of the NP only (c; which manifests itself as distortion of the NP surface close to the CO) contribute roughly equally to the energies. In principle finding a minimum energy path over the entire range of distances can be done, by performing a constrained relaxation at every distance. Such a calculation would yield a curve that is at every distance lower in energy than any other curve, effectively widening the adsorption minimum. Note that as the reference $E_{\text{NP}}^{(\text{site})}$, and $E_{\text{mol}}^{(\text{site})}$ are taken for the fully relaxed configurations binding energies at long distances are negative for options (b-d); this is the expected behavior for our definition of binding energy.

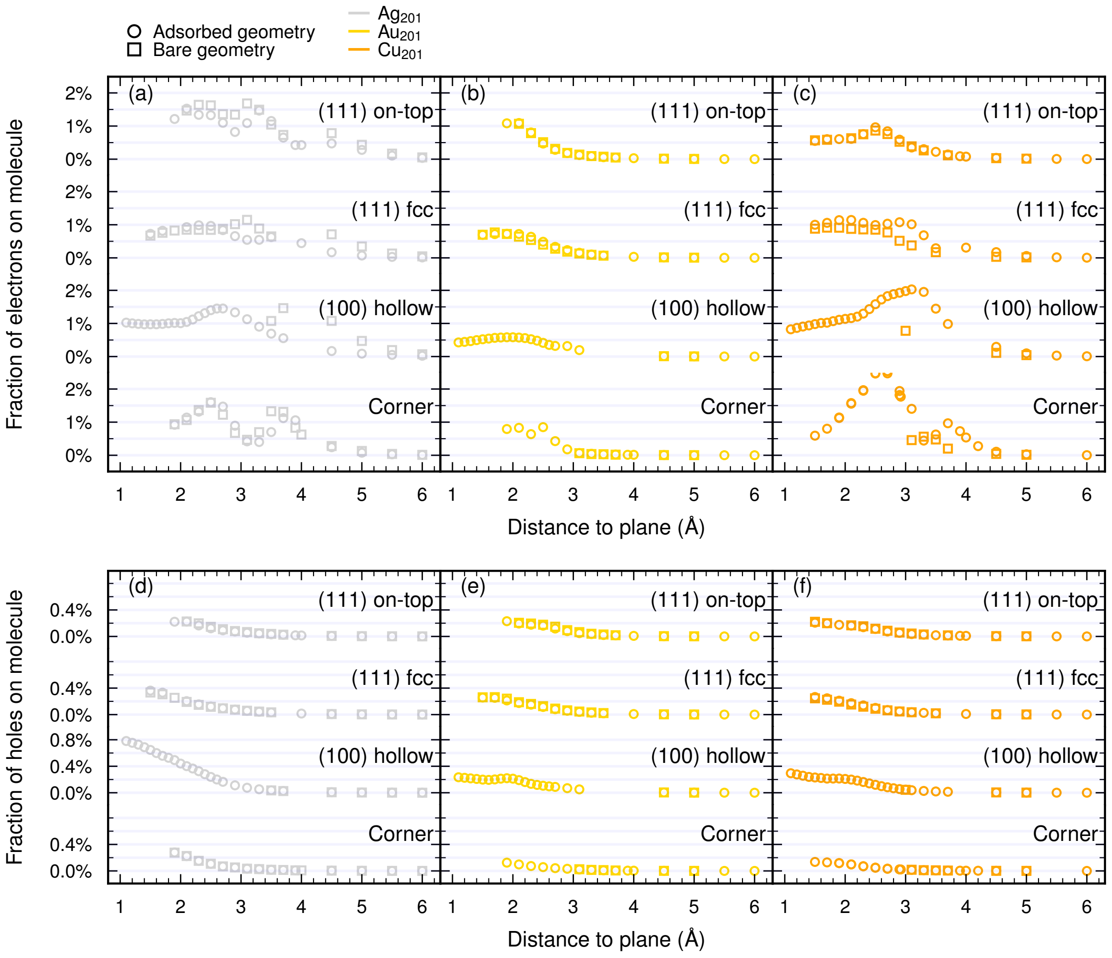

Figure S11: **Carrier generation on the molecule after plasmon decay.** Electrons (a-c) and holes (d-f) generated in Ag_{201} (a, d), Au_{201} (b, e), Cu_{201} (c, f) for the considered sites, distances, and geometrical configurations. "Adsorbed geometry" refers to the combined system that has been relaxed in the adsorption minimum, and the molecule then shifted. "Bare geometry" refers to the NP being configured as the free NP and the CO with the bond length of the free CO molecule. For the two types of geometries, the curves are similar for the electrons (with the exception of a few features), and practically identical for the holes. The differences for the electrons are explained by the shift in the CO LUMO orbital due to changing bond length, which affects the resonance condition (Fig. S9). Pulse frequencies are 3.8 eV (Ag), 2.5 eV (Au) and 2.7 eV (Cu). The fractions of generated carriers are computed as the average between 25 and 30 fs in the simulation.

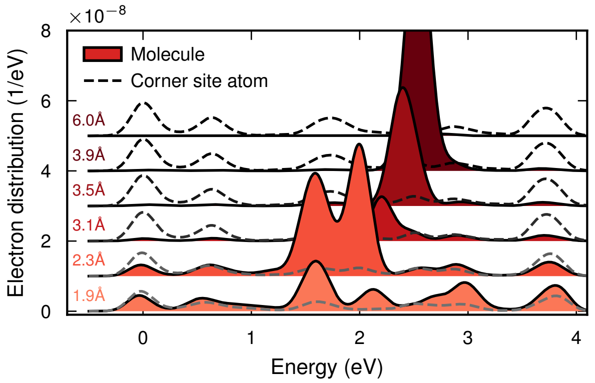

Figure S12: **Energy distribution of electrons generated on the molecule and on the NP corner site that the molecule approaches.** While the former varies non-monotonically, the latter is practically unchanged with distance.

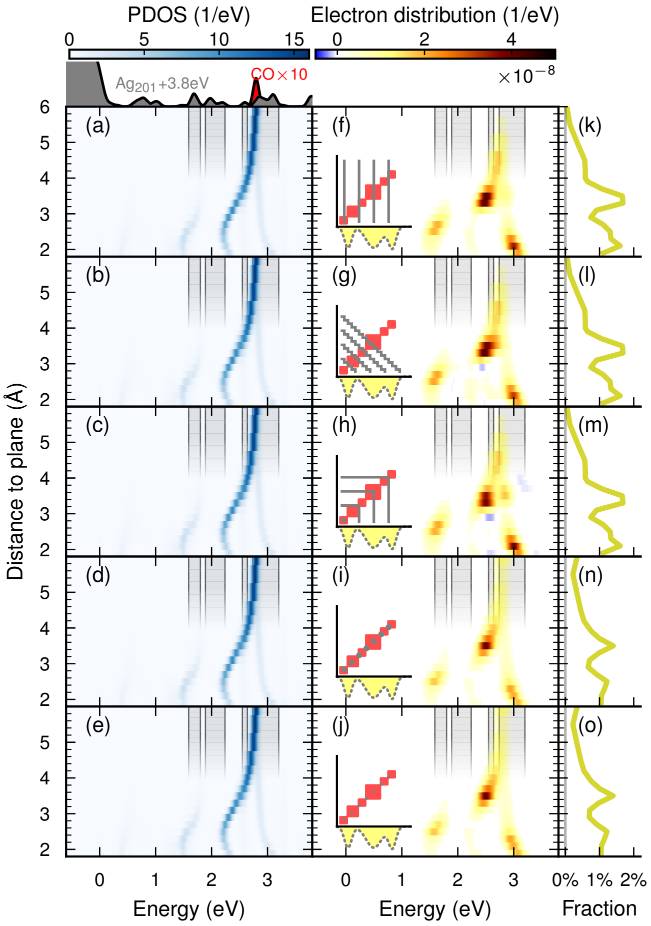

Figure S13: **Alternative formulations of electron distribution in molecule after plasmon decay.** The electron distribution in the molecule is well defined as a sum over unoccupied-unoccupied space $P_{\mathrm{e}}^{(\mathrm{mol})}(\boldsymbol{r}) = \frac{1}{2}\sum_{aa'} M_{aa'} w_{aa'}^{(\mathrm{mol})}$ of the density matrix product $M_{aa'} = \sum_i^{f_i > f_a, f_i > f_{a'}} (q_{ia}q_{ia'} + p_{ia}p_{ia'})$, $w_{aa'}^{(\mathrm{mol})} = \int_{\mathrm{mol}} \psi_a^{(0)}(\boldsymbol{r})\psi_{a'}^{(0)}(\boldsymbol{r})\mathrm{d}\boldsymbol{r}$. Simultaneous resolution in energy is not unambiguously defined. (a-e) PDOS on the molecule as a function of distance. All panels are identical. (f-j) Electron distribution expressed as (f) row/column-wise summation $\sum_{aa'} M_{aa'} w_{aa'}^{(\mathrm{mol})} \delta(\varepsilon - \varepsilon_a)$. (g) energy average summation $\sum_{aa'} M_{aa'} w_{aa'}^{(\mathrm{mol})} \delta(\varepsilon - \frac{\varepsilon_a + \varepsilon_{a'}}{2})$. (h) wedge-shape summation $\sum_{aa'} M_{aa'} w_{aa'}^{(\mathrm{mol})} \delta(\varepsilon - \max(\varepsilon_a, \varepsilon_{a'}))$. (i) degenerate-eigenvalue summation $\sum_{aa'} M_{aa'} w_{aa'}^{(\mathrm{mol})} \delta(\varepsilon - \varepsilon_a)\delta(\varepsilon_a - \varepsilon_{a'})$. (j) diagonal-only summation $\sum_{aa'} M_{aa'} w_{aa'}^{(\mathrm{mol})} \delta(\varepsilon - \varepsilon_a)\delta_{aa'}$. Note that between the forms (f-h) the distribution varies only slightly, however with some negative (unphysical) contributions to (g-h). (k-o) Energy integral of the distributions (f-j). For all but the diagonal-only and degenerate eigenvalues summation the end results are identical. The electron distributions are computed at 30 fs in the simulation for the Ag_{201} (111) on-top configuration.

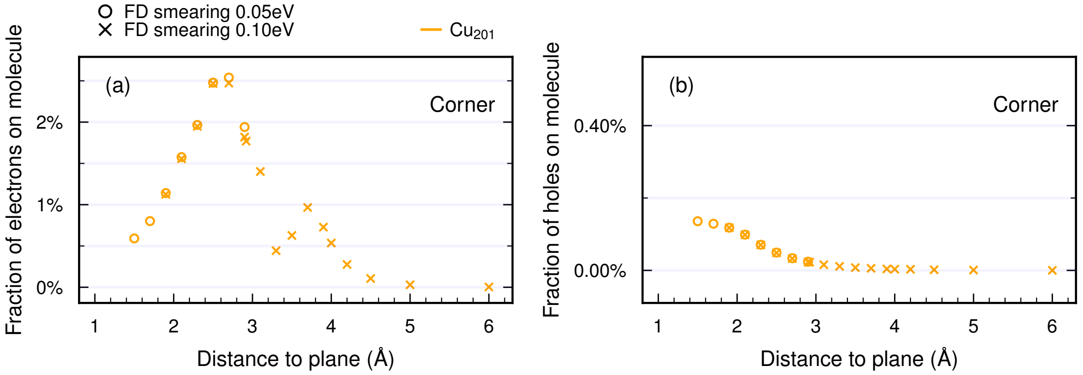

Figure S14: **Carrier generation depending on occupation number smearing.** We compare the fraction of electrons (a) and holes (b) generated on the molecule for the corner site of the Cu_{201} NP. Fermi-Dirac occupation number smearing with the parameters 0.05 eV and 0.1 eV was used. Data is not available at certain distances for the lower value of the smearing parameter (as SCF cycle convergence was difficult to reach with the GLLB-SC xc-functional) but is otherwise in good agreement with the higher parameter value. The fractions of electrons are computed as the average between 25 and 30 fs in the simulation for the Ag_{201} (111) on-top configuration.

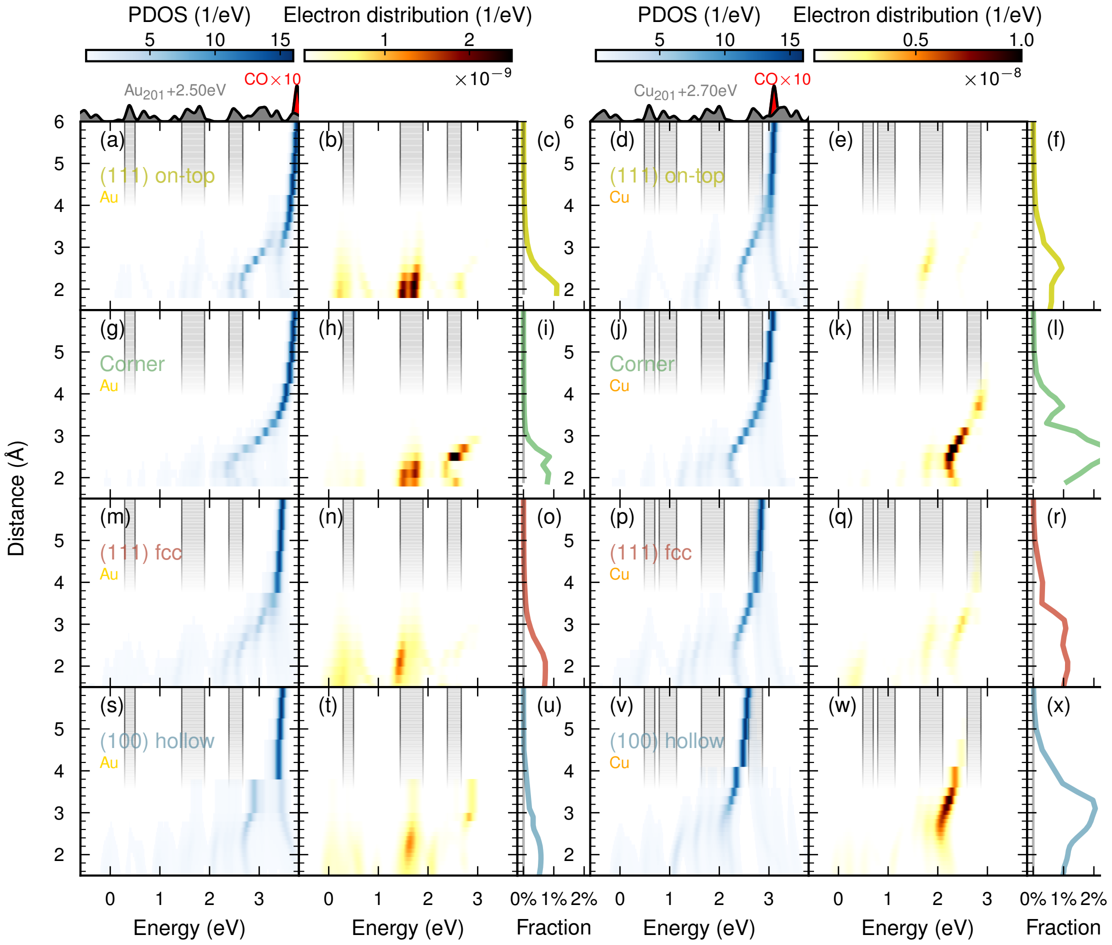

Figure S15: **Level alignment between the projected densities of state of the NP and molecule for Au_{201} and Cu_{201}.** The electron distributions are computed as the average between 25 and 30 fs in the simulation for the Ag_{201} (111) on-top configuration.

Supplementary Tables

Table S1: **Summary of binding energies, vibrational frequencies and associated bond distances.** Vibrational frequencies of the metal-C bond were computed by fitting a Morse potential to the binding energy curves (Fig. S7). Binding energies are here given as positive quantities $E_{\mathrm{NP}} + E_{\mathrm{mol}} - E_{\mathrm{NP+mol}}$. Data computed in this work, in other theoretical work (Ref. 1) and from experiment (Refs. 2, 3 and 4) is presented. The agreement is good where data is available.
TW: This work. Lit.: Literature.

	C-O distance (Å)		Metal-C distance (Å)		Metal-C frequency (cm^{-1})		Binding energy (meV)	
**Ag_{201}**	TW	Lit.	TW	Lit.	TW	Lit.	TW	Lit.
(111) on-top	1.151	1.150[1]	2.1	2.2[1]	203	203[1]	295	160[1]
(111) fcc	1.170	1.168[1]	1.6	1.6[1]	176		215	80[1]
(100) hollow	1.184		1.4		163		356	
corner	1.150		2.1		250		531	
**Au_{201}**								
(111) on-top	1.148	1.150[1]	2.0	2.1[1]	244		351	320[1]
(111) fcc	1.180	1.178[1]	1.4	1.4[1]	245		316	300[1]
(100) hollow	1.176		1.5		225		522	
corner	1.152		2.0		347		894	
**Cu_{201}**								
(111) on-top	1.158	1.156[1]	1.8	2.0[1]/1.9[2]	338	323[1]/346[3]/331[4]	787	750[1]
(111) fcc	1.183	1.179[1]	1.4	1.4[1]	286		929	880[1]
(100) hollow	1.205		1.2		232		1058	
corner	1.155		1.8		355		1002	

Supplementary Notes

Supplementary Note S1: Geometry of atomic structures

In this work we study the regular truncated octahedron (RTO)-shaped Ag_{201}, Au_{201}, and Cu_{201} nanoparticles (NPs). The RTO shape is consistent with an ideal face-centered cubic (fcc) lattice that has been truncated so that it has eight {111} surfaces and six {100} surfaces. We identify four molecular adsorption sites on the NP. On the (111) surface we consider the center on-top site and its nearest fcc site. We also consider the hollow site closest to the center of the (100) face, and the corner site between a {100} and two {111} surfaces. In each site the immediate environment of the molecule is different; it has one nearest-neighbor metal atom for the (111) on-top and corner sites, three for the (111) fcc site and four for the (100) hollow site.

We place a CO molecule (C facing the metal) near each site and find minimum energy configurations by allowing all metal and molecule atoms to relax. In the relaxed configurations (Table S1) the bond length and NP-molecule distance are thus different for each site. The bond length of CO, which is 1.144 Å in the free molecule, increases when adsorbed to the NP. Higher coordination numbers for the C atom thus result in larger bond lengths. This is in agreement with previous studies on the extended (111) Ag surface[1], and can be understood by considering that as C shares more electron density in bonds with metal atoms, the CO bond is weakened.

We study the distance dependence by rigidly shifting the molecule from the adsorbed configuration, i.e., without changing the CO bond length or the positions of the metal atoms. The shift is performed along a line perpendicular to the respective surface for the (111) and (100) sites and along the line through the opposing corner site for the corner site. We note that at long separations such rigidly shifted configurations are relatively high in energy. Specifically, at long distances the minimum energy configuration would be a molecule with the bond length of bare CO and a fully relaxed bare NP. In general, finding such minimum energy configurations under a distance constraint is a highly multidimensional problem. We therefore limit our discussion to rigid translations of the molecule, without compromising our conclusions (Fig. S9).

As we vary the NP-CO distance, we observe minima in the binding energy curves around 250 meV to 500 meV at 1.5 Å-2.3 Å (Fig. S7). Distances corresponding to energy minima as well as the computed vibrational frequencies (Table S1) are in good agreement with earlier calculations[1] and experiment[2–4]. Here, we define the binding energy as

$$E_{\mathrm{bind}}^{(\mathrm{site})}(d) = E^{(\mathrm{site})}(d) - E_{\mathrm{NP}}^{(\mathrm{site})} - E_{\mathrm{mol}}^{(\mathrm{site})}, \tag{S1}$$

where $E_{\mathrm{NP}}^{(\mathrm{site})}$ and $E_{\mathrm{mol}}^{(\mathrm{site})}$ are the energies of the NP and molecule, respectively, taken from two separate calculations, representing infinite separation. Note that in this definition (Eq. S1), we take $E_{\mathrm{NP}}^{(\mathrm{site})}$ and $E_{\mathrm{mol}}^{(\mathrm{site})}$ as the energies of the NP and molecule as in the relaxed configuration for the specific site, emphasized by the superscript (site). Allowing the molecule to relax at each distance effectively widens the adsorption curve but does not affect the main conclusions drawn in our work (Fig. S10, Fig. S11). Fixing the bond length reduces, however, the degrees of freedom and simplifies the discussion, whence we adopt this constraint here.

In our work we find that the hot carrier (HC) transfer landscape has a detailed structure. It is then important to remember that in reality both the molecule and the NP are subjected to thermal motion. Since it is the weakest interaction in the combined system, one can expect the relative motion of NP and molecule to have the most pronounced thermal effect on level alignment, effectively broadening the peaks in the HC transfer probability curves. Considering $kT \approx 25$ meV at room temperature and the calculated binding energy curves (Fig. S7), we should expect the molecule to move much less than 1 Å along the distance axis, but in reality the molecule is free to move laterally as well as rotate. Sampling the landscape of HC transfer in the full space of molecular movement is a high-dimensional problem that can be addressed in future work.

Supplementary Note S2: Comparison of across-interface electron generation to surface electron distribution

We emphasize that the energy distribution of electrons generated on the molecule depends on energetic level alignment of the molecular levels, by computing the energy distribution of electrons on the nearest metal atom (the integral of Eq. S22 in the metal-atom Voronoi cell) as a function of distance (Fig. S12). We focus specifically on the corner site, where the distance dependence of the electron distribution on the molecule exhibits two clear maxima. We find that the electron distribution on the adsorption site is practically distance-independent.

Only at the smallest considered distances does the electron distribution on the molecule resemble the electron distribution at the adsorption site on the metal. Hence it is not enough to know the electron distribution on surfaces and surface sites for a bare NP to predict across-interface electron generation in combined NP + molecule systems. Equipped with distributions for the bare NP alone one misses for example that about 2 times more electrons are generated at 2.5 Å for the corner site, than at 1.9 Å. In other words, the surface electron distribution is an insufficient predictor for across-interface electron generation.

Supplementary Note S3: Methodology

In the limit of weak perturbation, the system response can be assumed to be linear. This means that the Fourier transform of the *response* at one particular frequency depends only on the Fourier transform of the *perturbation* at the same frequency, and the linear response to a time-dependent perturbation can be retrieved knowing the linear impulse response. Similarly to Ref. 5, we use this property in real-time time-dependent density functional theory (RT-TDDFT) to compute optical spectra and model hot-carrier generation in arbitrary weak electric fields. For simplicity we adopt Hartree atomic units in this section.

Plasmonic response in the δ-kick technique

In the δ-kick technique[6] the time-dependent external potential is set to a kick in the z-direction of strength K_z

$$v_{\text{ext}}(\boldsymbol{r}, t) = K_z z \delta(t), \tag{S2}$$

and the system is allowed to evolve in time.

We define the induced density due to the δ-kick $\delta n^{\text{kick}}(\boldsymbol{r}, t) = n^{\text{kick}}(\boldsymbol{r}, t) - n(\boldsymbol{r}, 0)$ and the induced dipole moment in the z-direction as

$$\delta\mu_z^{\text{kick}}(t) = \mu_z^{\text{kick}}(t) - \mu_z(0) = \int \mathrm{d}\boldsymbol{r} \delta n^{\text{kick}}(\boldsymbol{r}, t) z. \tag{S3}$$

The optical spectrum in the z direction can then be expressed via the dipole strength function

$$S_z(\omega) = -\frac{2\omega}{\pi} \Im \left[\frac{\mu_z(\omega)}{K_z} \right], \tag{S4}$$

where $\mu_z(\omega)$ is the Fourier transform of $\mu_z(t)$.

Carrier generation in the δ-kick technique

In Kohn-Sham (KS)-RT-TDDFT the KS density operator

$$\rho(t) = \sum_k |\phi_k(t)\rangle f_k \langle\phi_k(t)| \tag{S5}$$

contains all information about the system. Here, $\phi_k(t)$ are the KS wave functions at time t and f_k their ground state occupation numbers. In practice, the operator is expressed as the KS density matrix

in the basis of ground state wave functions $|\phi_k^{(0)}\rangle = |\phi_k(0)\rangle$

$$\rho_{nn'}(t) = \langle\phi_n^{(0)}|\,\rho(t)\,|\phi_{n'}^{(0)}\rangle \tag{S6}$$

$$= \sum_k \langle\phi_n^{(0)}|\,|\phi_k(t)\rangle\, f_k\, \langle\phi_k(t)|\,|\phi_{n'}^{(0)}\rangle\,. \tag{S7}$$

In linear response we can express the KS density matrix $\rho_{nn'}(t)$ corresponding to an arbitrary time-dependent field $\boldsymbol{\mathcal{E}}(t)$ in terms of the KS density matrix $\rho_{nn'}^{\text{kick}}(t)$ due to the δ-kick

$$\rho_{nn'}(\omega) = \frac{1}{K_z}\rho_{nn'}^{\text{kick}}(\omega)\mathcal{E}_z(\omega). \tag{S8}$$

Here, functions of ω are Fourier transforms of the corresponding time-dependent quantities, and we have constrained ourselves to fields where the z-component $\mathcal{E}_z$ is the only non-zero component of $\boldsymbol{\mathcal{E}}$. By the convolution theorem Eq. S8 is equivalent to

$$\rho_{nn'}(t) = \frac{1}{K_z}\int_0^\infty \mathrm{d}\tau \rho_{nn'}^{\text{kick}}(\tau)\mathcal{E}_z(t-\tau). \tag{S9}$$

In practice, we compute the Fourier transform of the KS density matrix during the time propagation with the δ-kick and obtain the response to other fields as a post-processing step, using Eq. S8 and inverse Fourier transformation.

To compute the distributions of generated carriers we follow the method of Ref. 5, where expressions that include second-order corrections to the density matrix are derived. For notational convenience (see Supplementary Note 2 of Ref. 5), we introduce the notation

$$q_{ia}(t) = 2\Re\delta\rho_{ia}(t)\big/\sqrt{2(f_i - f_a)} \tag{S10}$$

$$p_{ia}(t) = -2\Im\delta\rho_{ia}(t)\big/\sqrt{2(f_i - f_a)}, \tag{S11}$$

which is well-defined for matrix elements corresponding to occupied (i) – unoccupied (a) pairs, $f_i > f_a$. Only these occupied–unoccupied pairs are needed to construct the observables of interest.

The probability that a hole (electron) has been generated at time t in state i (a) is

$$P_i^{\text{h}}(t) = \sum_a^{f_i > f_a} \frac{1}{2}\left[q_{ia}^2(t) + p_{ia}^2(t)\right] \tag{S12}$$

$$P_a^{\text{e}}(t) = \sum_i^{f_i > f_a} \frac{1}{2}\left[q_{ia}^2(t) + p_{ia}^2(t)\right]. \tag{S13}$$

The sum of generated carriers is the same for both types

$$N_{\text{carriers}}(t) = \sum_i P_i^{\text{h}}(t) = \sum_a P_a^{\text{e}}(t). \tag{S14}$$

These probabilities are alternatively expressed as spatial probability densities (note that we drop the explicit t dependence in q and p for brevity)

$$P_{\text{h}}(\boldsymbol{r},t) = \frac{1}{2}\sum_{aii'}^{f_i>f_a, f_{i'}>f_a} (q_{ia}q_{i'a} + p_{i'a}p_{ia})\psi_i^{(0)}(\boldsymbol{r})\psi_{i'}^{(0)}(\boldsymbol{r}) \tag{S15}$$

$$P_{\text{e}}(\boldsymbol{r},t) = \frac{1}{2}\sum_{iaa'}^{f_i>f_a, f_i>f_{a'}} (q_{ia}q_{ia'} + p_{ia}p_{ia'})\psi_a^{(0)}(\boldsymbol{r})\psi_{a'}^{(0)}(\boldsymbol{r}). \tag{S16}$$

Note that integrating Eq. S15 and Eq. S16 over the entire space yields the number density of carriers $\int \mathrm{d}\boldsymbol{r}P^{\text{e/h}}(\boldsymbol{r},t) = N_{\text{carriers}}(t)$. Further, we construct spatio-energetic probability density contributions

$$P_{\text{h}}(\varepsilon,\boldsymbol{r},t) = \frac{1}{2}\sum_{aii'}^{f_i>f_a, f_{i'}>f_a} (q_{ia}q_{i'a} + p_{i'a}p_{ia})\psi_i^{(0)}(\boldsymbol{r})\psi_{i'}^{(0)}(\boldsymbol{r})\delta(\varepsilon-\varepsilon_i) \tag{S17}$$

$$P_{\text{e}}(\varepsilon,\boldsymbol{r},t) = \frac{1}{2}\sum_{iaa'}^{f_i>f_a, f_i>f_{a'}} (q_{ia}q_{ia'} + p_{ia}p_{ia'})\psi_a^{(0)}(\boldsymbol{r})\psi_{a'}^{(0)}(\boldsymbol{r})\delta(\varepsilon-\varepsilon_a). \tag{S18}$$

The δ-functions in energy are in practice approximated by a Gaussian $(2\pi\sigma^2)^{-1/2}\exp(-\varepsilon^2/2\sigma^2)$ with width $\sigma = 0.07\,\mathrm{eV}$. There is no unique definition for the simultaneous decomposition in time and energy but we obtain consistent results (Fig. S13) with different variants of the former expressions. Note that in contrast to Ref. 5 we include the summation of non-degenerate ii' and aa' which has significant implications in the NP+molecule system (Fig. S13).

In this work, we calculate the fraction of generated carriers in particular regions, i.e., the Voronoi region of the molecule or the Voronoi region of a particular atomic site

$$P^{\mathrm{h,region}}(t) = \int_{\mathrm{region}} \mathrm{d}\boldsymbol{r} P_{\mathrm{h}}(\boldsymbol{r}, t)/N_{\mathrm{carriers}}(t) \tag{S19}$$

$$P^{\mathrm{e,region}}(t) = \int_{\mathrm{region}} \mathrm{d}\boldsymbol{r} P_{\mathrm{e}}(\boldsymbol{r}, t)/N_{\mathrm{carriers}}(t). \tag{S20}$$

Similarly, we calculate the energetic distribution in a particular region

$$P^{\mathrm{h,region}}(\varepsilon, t) = \int_{\mathrm{region}} \mathrm{d}\boldsymbol{r} P_{\mathrm{h}}(\varepsilon, \boldsymbol{r}, t) \tag{S21}$$

$$P^{\mathrm{e,region}}(\varepsilon, t) = \int_{\mathrm{region}} \mathrm{d}\boldsymbol{r} P_{\mathrm{e}}(\varepsilon, \boldsymbol{r}, t). \tag{S22}$$

Supplementary Note S4: Computational details

The VASP[7–9] suite was used for all structure relaxations and total energy calculations. Ground state energies were computed using a plane-wave basis set, the projector augmented wave (PAW)[10,11] method, and the vdW-df-cx[12–15] exchange correlation (XC)-functional. The plane wave cutoff 500 eV was used. We used a Gaussian occupation number smearing scheme with the parameter 0.1 eV.

Structure relaxations were performed using the conjugate gradient relaxation method implemented in VASP. Relaxation was stopped when the maximal force on any atom fell below $0.015\,\mathrm{eV\,Å^{-1}}$. Relaxations were performed for each NP separately to obtain relaxed bare NP structures. Additionally for each NP and each adsorption site the NP+molecule system was relaxed, with all atoms allowed to move.

The open-source GPAW[16,17] code package was used for all calculations of plasmonic response and carrier generation. Kohn-Sham DFT (KS-DFT) ground state calculations were performed within the PAW[10] formalism using linear combination of atomic orbitals (LCAO) basis sets[18]; the double-ζ polarized (dzp) basis set was used for C and O, and the *pvalence*[19] basis set, which is optimized to represent bound unoccupied states, for Ag, Au and Cu. The Gritsenko-van Leeuwen-van Lenthe-Baerends-solid-correlation (GLLB-sc)[20,21] XC functional was used. A simulation cell of $32\,\mathrm{Å} \times 32\,\mathrm{Å} \times 38.4\,\mathrm{Å}$ was used to represent wave functions, XC, and Coulomb potentials, with a grid spacing of 0.2 Å for wave functions and 0.1 Å for potentials. The Coulomb potential was represented in numerical form on the grid, with an additional analytic moment correction[22] centered at the NP. Fermi-Dirac occupation number smearing with width 0.05 eV was used for all calculations, except for the Cu corner site for distances $\geq$2.7 Å and for Au (111) on-top distances between 3.1 and 3.7 Å. The effect of different occupation number smearing is negligible for our results (Fig. S14). The self-consistent loop was stopped when the integral of the difference between two subsequent densities was less than 1×10^{-8}. Pulay[23]-mixing was used to accelerate the ground state convergence.

The LCAO-RT-TDDFT implementation[19] in GPAW was used for the RT-TDDFT calculations. A δ-kick strength of $K_z = 10^{-5}$ in atomic units was used. The time propagation was done in steps of 10 as for a total length of 30 fs using the adiabatic GLLB-sc kernel. We computed carrier generation for an external electric field corresponding to an ultra-short Gaussian laser pulse

$$\mathcal{E}_z(t) = \mathcal{E}_0 \cos(\omega_0 t) \exp(-(t - t_0)^2/\tau_0^2) \tag{S23}$$

of frequency ω, strength $\mathcal{E}_0 = 51\,\mathrm{\mu V\,Å^{-1}}$, peak time $t_0 = 10\,\mathrm{fs}$, and duration $\tau_0 = 3.0\,\mathrm{fs}$.

We computed the total density of states (DOS) as

$$\sum_k \delta(\varepsilon - \varepsilon_k) \tag{S24}$$

and the molecular projected DOS (PDOS) as

$$\sum_k \delta(\varepsilon - \varepsilon_k) \int_{\text{molecule}} \left| \phi_k^{(0)}(\boldsymbol{r}) \right|^2 \mathrm{d}\boldsymbol{r}, \tag{S25}$$

where ε_k and $\phi_k^{(0)}(\boldsymbol{r})$ are the KS eigenvalues and wave functions. For visualization, the δ-functions in energy were replaced by a Gaussian $(2\pi\sigma^2)^{-1/2} \exp(-\varepsilon^2/2\sigma^2)$ with width $\sigma = 0.05\,\mathrm{eV}$.

Supplementary References

[1] Marek Gajdo, Andreas Eichler, and Jürgen Hafner. CO adsorption on close-packed transition and noble metal surfaces: Trends from *ab initio* calculations. *Journal of Physics: Condensed Matter*, 16(8):1141–1164, 2004. doi: 10.1088/0953-8984/16/8/001.

[2] Edward J. Moler, Scot A. Kellar, W. R. A. Huff, Zahid Hussain, Yufeng Chen, and David A. Shirley. Spatial structure determination of $(\sqrt{3} \times \sqrt{3})$r30° and (1.5×1.5)r18° co or cu(111) using angle-resolved photoemission extended fine structure. *Physical Review B*, 54(15):10862–10868, 1996. doi: 10.1103/PhysRevB.54.10862.

[3] C. J. Hirschmugl, G. P. Williams, F. M. Hoffmann, and Y. J. Chabal. Adsorbate-substrate resonant interactions observed for Co on Cu(100) and (111) in the far-ir using synchrotron radiation. *Journal of Electron Spectroscopy and Related Phenomena*, 54-55:109–114, 1990. ISSN 0368-2048. doi: 10.1016/0368-2048(90)80203-M.

[4] R. Raval, S. F. Parker, M. E. Pemble, P. Hollins, J. Pritchard, and M. A. Chesters. FT-rairs, eels and leed studies of the adsorption of carbon monoxide on Cu(111). *Surface Science*, 203(3): 353–377, 1988. ISSN 0039-6028. doi: 10.1016/0039-6028(88)90088-X.

[5] Tuomas P. Rossi, Paul Erhart, and Mikael Kuisma. Hot-Carrier Generation in Plasmonic Nanoparticles: The Importance of Atomic Structure. *ACS Nano*, 14(8):9963–9971, 2020. doi: 10.1021/acsnano.0c03004.

[6] K. Yabana and G. F. Bertsch. Time-dependent local-density approximation in real time. *Physical Review B*, 54(7):4484–4487, 1996. doi: 10.1103/PhysRevB.54.4484.

[7] G. Kresse and J. Hafner. Ab initio molecular dynamics for liquid metals. *Physical Review B*, 47 (1):558–561, 1993. doi: 10.1103/PhysRevB.47.558.

[8] G. Kresse and J. Furthmüller. Efficient iterative schemes for ab initio total-energy calculations using a plane-wave basis set. *Physical Review B*, 54(16):11169–11186, 1996. doi: 10.1103/PhysRevB.54.11169.

[9] G. Kresse and J. Furthmüller. Efficiency of ab-initio total energy calculations for metals and semiconductors using a plane-wave basis set. *Computational Materials Science*, 6(1):15–50, 1996. doi: 10.1016/0927-0256(96)00008-0.

[10] P. E. Blöchl. Projector augmented-wave method. *Physical Review B*, 50(24):17953–17979, 1994. doi: 10.1103/PhysRevB.50.17953.

[11] G. Kresse and D. Joubert. From ultrasoft pseudopotentials to the projector augmented-wave method. *Physical Review B*, 59(3):1758–1775, 1999. doi: 10.1103/PhysRevB.59.1758.

[12] M. Dion, H. Rydberg, E. Schröder, D. C. Langreth, and B. I. Lundqvist. Van der Waals Density Functional for General Geometries. *Physical Review Letters*, 92(24):246401, 2004. doi: 10.1103/PhysRevLett.92.246401.

[13] Kristian Berland and Per Hyldgaard. Exchange functional that tests the robustness of the plasmon description of the van der Waals density functional. *Physical Review B*, 89(3):035412, 2014. doi: 10.1103/PhysRevB.89.035412.

[14] Jiří Klimeš, David R. Bowler, and Angelos Michaelides. Chemical accuracy for the van der Waals density functional. *Journal of Physics: Condensed Matter*, 22(2):022201, 2009. doi: 10.1088/0953-8984/22/2/022201.

[15] Guillermo Román-Pérez and José M. Soler. Efficient Implementation of a van der Waals Density Functional: Application to Double-Wall Carbon Nanotubes. *Physical Review Letters*, 103(9):096102, 2009. doi: 10.1103/PhysRevLett.103.096102.

[16] J. J. Mortensen, L. B. Hansen, and K. W. Jacobsen. Real-space grid implementation of the projector augmented wave method. *Physical Review B*, 71(3):035109, 2005. doi: 10.1103/PhysRevB.71.035109.

[17] J Enkovaara, C Rostgaard, J J Mortensen, J Chen, M Dułak, L Ferrighi, J Gavnholt, C Glinsvad, V Haikola, H A Hansen, H H Kristoffersen, M Kuisma, A H Larsen, L Lehtovaara, M Ljungberg, O Lopez-Acevedo, P G Moses, J Ojanen, T Olsen, V Petzold, N A Romero, J Stausholm-Møller, M Strange, G A Tritsaris, M Vanin, M Walter, B Hammer, H Häkkinen, G K H Madsen, R M Nieminen, J K Nørskov, M Puska, T T Rantala, J Schiøtz, K S Thygesen, and K W Jacobsen. Electronic structure calculations with GPAW: A real-space implementation of the projector augmented-wave method. *Journal of Physics: Condensed Matter*, 22(25):253202, 2010. doi: 10.1088/0953-8984/22/25/253202.

[18] A. H. Larsen, M. Vanin, J. J. Mortensen, K. S. Thygesen, and K. W. Jacobsen. Localized atomic basis set in the projector augmented wave method. *Physical Review B*, 80(19):195112, 2009. doi: 10.1103/PhysRevB.80.195112.

[19] M. Kuisma, A. Sakko, T. P. Rossi, A. H. Larsen, J. Enkovaara, L. Lehtovaara, and T. T. Rantala. Localized surface plasmon resonance in silver nanoparticles: Atomistic first-principles time-dependent density-functional theory calculations. *Physical Review B*, 91(11):115431, 2015. doi: 10.1103/PhysRevB.91.115431.

[20] Oleg Gritsenko, Robert van Leeuwen, Erik van Lenthe, and Evert Jan Baerends. Self-consistent approximation to the Kohn-Sham exchange potential. *Physical Review A*, 51(3):1944, 1995. doi: 10.1103/PhysRevA.51.1944.

[21] M. Kuisma, J. Ojanen, J. Enkovaara, and T. T. Rantala. Kohn-Sham potential with discontinuity for band gap materials. *Physical Review B*, 82(11):115106, 2010. doi: 10.1103/PhysRevB.82.115106.

[22] A Castro, A Rubio, and M J Stott. Solution of Poisson's equation for finite systems using plane-wave methods. *Canadian Journal of Physics*, 81(10):1151–1164, 2003. doi: 10.1139/p03-078.

[23] Péter Pulay. Convergence acceleration of iterative sequences. the case of scf iteration. *Chemical Physics Letters*, 73(2):393–398, 1980. doi: 10.1016/0009-2614(80)80396-4.
